# Faecalcrobiota metabolites: emerging insights into cancer radiotherapy outcomes

**DOI:** 10.3389/fmicb.2025.1663835

**Published:** 2025-09-18

**Authors:** Yuzhuo Gao, Baozhao Zeng, Zhicheng Wang, Shuo Liang, Yanming Yang

**Affiliations:** ^1^Department of Radiotherapy, The Second Hospital of Jilin University, Changchun, China; ^2^National Health Commission (NHC) Key laboratory of Radiobiology, School of Public Health, Jilin University, Changchun, China

**Keywords:** gut microbiota, metabolites, cancer, radiotherapy, radiation injury

## Abstract

The connection between gut microbiota and the onset, progression, and management of cancer is receiving increasing attention. Gut microbiota metabolites serve as crucial mediators that influence the cancer process by modulating immune responses and metabolic pathways. Research has shown that these metabolites significantly affect cancer development, prognosis and therapy. For example, the effectiveness and side effects of radiotherapy are closely linked to the metabolites produced by gut microbiota. Radiotherapy can disrupt the balance of gut microbiota, increase intestinal permeability, and trigger inflammatory responses, all of which may lead to adverse reactions such as damage to the intestinal mucosa and a compromised anti-cancer effect. This review emphasizes the role of gut microbiota metabolites in tumor formation and progression by affecting signaling pathways and the tumor immune microenvironment. It explores how these metabolites can influence the efficacy and side effects of radiotherapy and discusses innovative cancer treatment strategies that leverage gut microbiota metabolites. By integrating recent preclinical and clinical findings, the review proposes that incorporating colony modulation therapies into cancer treatment could enhance therapeutic strategies and provide patients with safer and more effective options.

## Graphical Abstract

**Figure:**
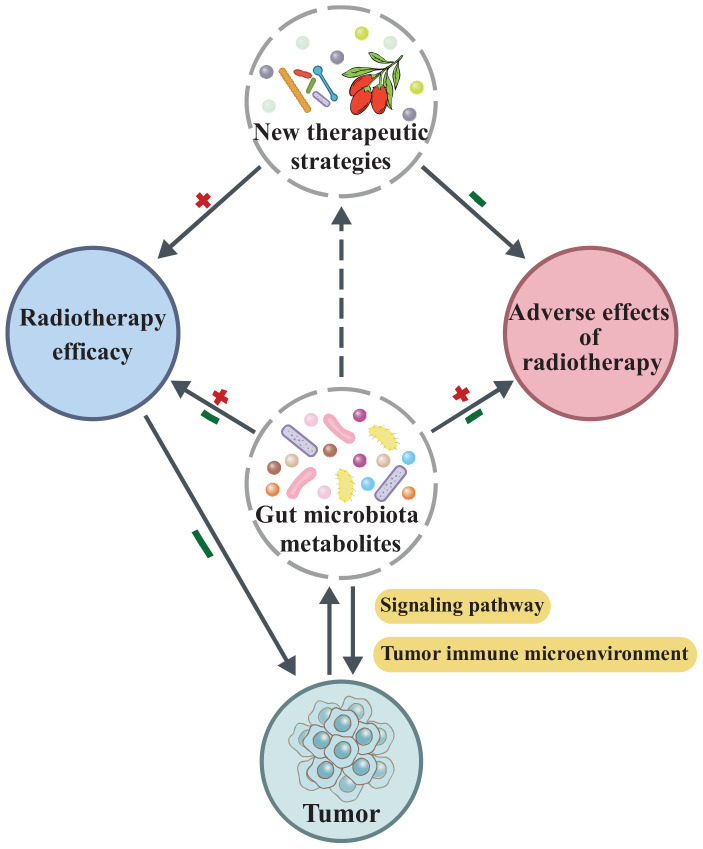
Gut microbial metabolites play a multifunctional role in oncogenesis and cancer therapy by not only modulating key signaling pathways and reshaping the tumor immune microenvironment, which influences tumor initiation and progression, but also by altering radiotherapy efficacy and mitigating treatment-related adverse effects. Moreover, these metabolites provide a foundation for developing novel therapeutic strategies aimed at enhancing radiotherapeutic outcomes while reducing toxicity.

## Highlights

- Alterations in gut microbiota and its metabolites have been associated with the development of several cancers.
- Gut microbiota metabolites influence tumor development and radiotherapy by modulating various signaling pathways.
- Gut microbiota metabolites can be used as predictors of response to radiotherapy, targeted therapy and immunotherapy.
- Modulation of gut microbiota metabolites through novel strategies can improve the efficacy of radiotherapy and mitigate adverse effects.

## 1 Introduction

Cancer is one of the leading causes of death worldwide. In recent years, numerous studies have shown evidence that gut microbiota is a key determinant of health or pathological conditions (Zhang et al., 2015). The predominant groups of human gut microbiota are the Bacteroidota and Firmicutes (Wroblewski et al., 2016). These gut microbiota generate antimicrobial compounds, defend against harmful microorganisms, encourage the growth of epithelial cells, and help preserve the integrity of the intestinal lining (Peng et al., 2020). Therefore, when the gut microbiota is imbalanced, its components change, diversity is reduced and metabolism is altered, leading to various inflammatory conditions and even cancers. Zitvogel et al. (2017) summarize the mechanisms by which gut microbiota promotes tumorigenesis, including through the direct oncogenic effects of microorganisms and their products. For instance, pathogenic pks + E. coli generates a genotoxin known as E. coli genotoxin, which can alkylate DNA and may play a role in the onset and advancement of colorectal cancer (Pleguezuelos-Manzano et al., 2020; Wilson et al., 2019). Furthermore, gut microbiota can generate a range of small molecules and metabolites, including short-chain fatty acids (SCFAs) and secondary bile acids, which may affect tumor growth through different mechanisms (Chen et al., 2023). The primary pathways involve altering inflammatory and immune responses within the tumor microenvironment (TME) and modifying signaling pathways that impact the expression of genes related to cancer (Yang et al., 2023). For example, lithocholic acid and deoxycholic acid (DCA), which are secondary bile acids, can induce colorectal cancer (CRC) by activating the G protein-coupled bile acid receptor (Chen et al., 2023). Due to the wide range of roles of microbial metabolites in cancer, more and more experiments are starting to explore them as targets for novel cancer therapies.

Radiotherapy is a conventional form of cancer treatment and is curative for 25% of cancers. However, radiation can cause tremendous damage to normal rapidly dividing cells, particularly the gastrointestinal epithelium, leading to acute enteropathy. Lu et al. (2024b) discovered that ^131^I radiotherapy notably changed the gut microbiota structure and metabolite composition in patients with differentiated thyroid cancer. They noted a reduction in the levels and pathways of metabolites associated with arachidonic acid (ARA) and linoleic acid (LA) following radiotherapy. Additionally, they found that supplementing with ARA not only enhanced quality of life and helped restore the hematopoietic and gastrointestinal systems, but also reduced oxidative stress and inflammation while maintaining the intestinal microecological balance (Lu et al., 2024b). In addition, germ-free and antibiotic-pretreated mice were less sensitive to radiation and exhibited less radiation-induced intestinal damage, suggesting a relationship between gut microbiota-derived metabolites and radiation-induced injury and damage repair (Zhao et al., 2020). Currently, SCFAs, aromatic amino acids, and bile acids have been identified as signaling molecules in the microbe-host dialogue that are involved in the regulation of host physiological functions under radiation conditions (Liu et al., 2023; Guo et al., 2023).

This article reviews the molecular mechanisms underlying the carcinogenic effects of gut microbiota metabolites, including regulatory signaling pathways and control of the tumor immune microenvironment. Based on this, it explores the significance of microbial metabolites in radiotherapy, including their impact on treatment efficacy and side effects. Additionally, it highlights emerging microbial intervention strategies and their clinical applications, with a focus on recent studies investigating how microbial metabolites can enhance the efficacy of radiotherapy while reducing its side effects. These findings may provide insights for developing personalized treatment strategies for cancer.

## 2 Gut microbiota-derived metabolites in cancer progression

### 2.1 Modulation-related signaling systems

**FIGURE 1:**
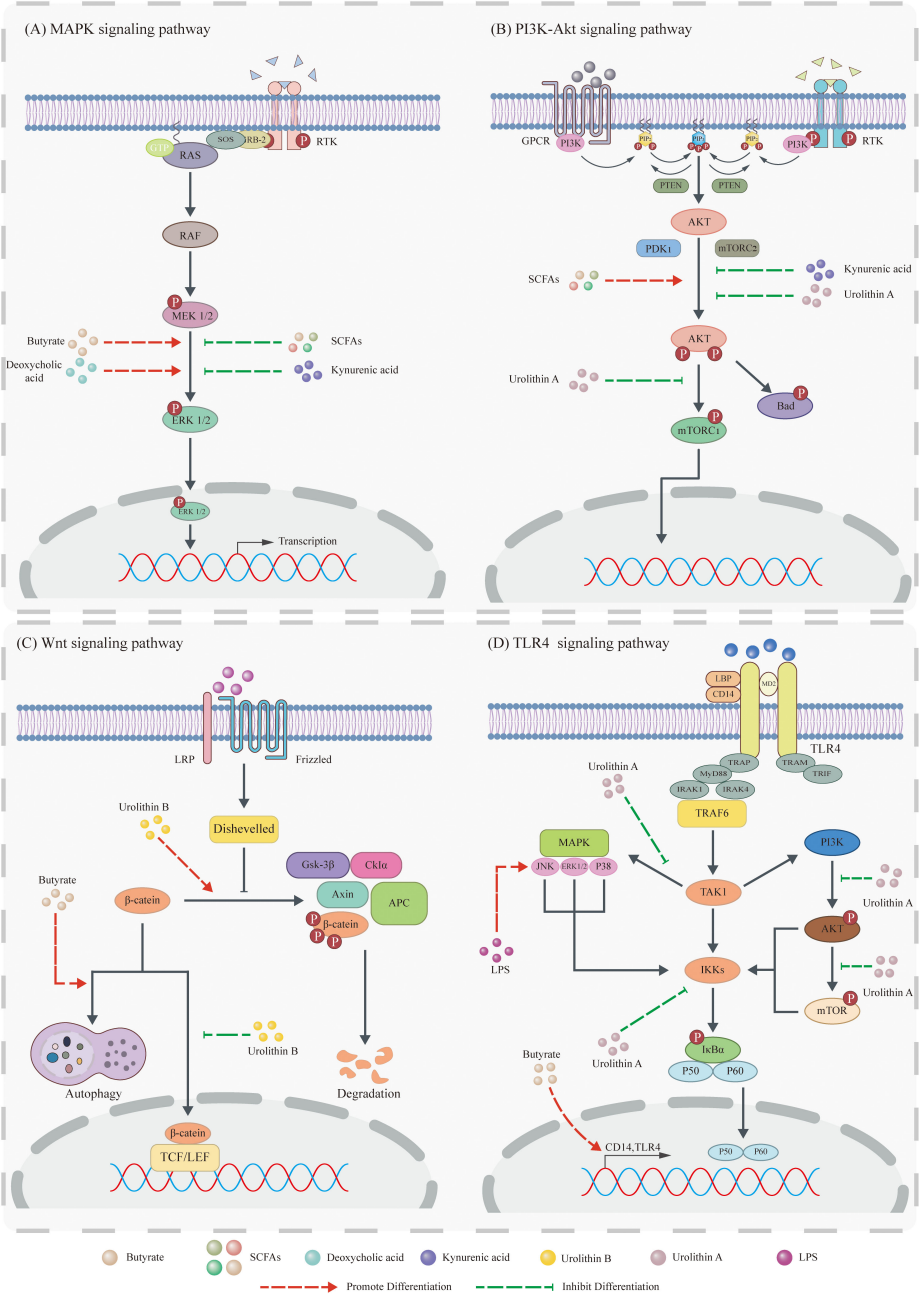
Gut microbiota metabolites influence tumor development by modulating signaling pathways. **(A)** Mitogen-activated protein kinase (MAPK) pathway: Butyrate and DCA activate the MAPK pathway by promoting Extracellular regulating kinase (ERK) phosphorylation, whereas SCFAs and Kynruenic acid (KYNA) suppress this pathway by inhibiting ERK phosphorylation. **(B)** PI3K pathway: SCFAs activate the PI3K pathway via Protein Kinase B (Akt) phosphorylation. In contrast, KYNA inhibits it by suppressing Akt phosphorylation, and Uro A downregulates it through inhibition of both Akt phosphorylation and mammalian target of rapamycin complex 1 (mTORC1) complex activity. **(C)** Wnt pathway: Butyrate inhibits Wnt pathway activation by degrading β-catenin via the autophagy-lysosomal pathway, while Urolithin B (Uro B) promotes its degradation through the proteasomal pathway. Additionally, Uro B suppresses β-catenin nuclear translocation to further inhibit this pathway. **(D)** TLR4 pathway: LPS activates the downstream JNK pathway via TLR4 signaling. Uro A collectively inhibits the TLR4 pathway by: (1) blocking phosphorylation of ERK, p38, and JNK (MAPKs), (2) inhibiting phosphorylation of Akt and mTOR proteins, and (3) reducing Inhibitor of NF-κB kinase (IKK) activity.

Signaling pathways serve as critical conduits for cellular communication, regulating essential processes including cell growth, differentiation, apoptosis, and metabolism through specific signaling molecules. While maintaining physiological homeostasis, their dysregulation frequently contributes to diseases such as tumorigenesis. Notably, gut microbial metabolites modulate these pathways to influence tumor cell invasion, metastasis, and proliferation (Figure 1 and Table 1).

**TABLE 1:** Multifaceted regulation of oncogenic signaling pathways by gut microbiota-derived metabolites.

Signaling pathway	Gut microbiota-derived metabolite	Cell/animal model	Key targets/mechanisms	Signaling pathway modulation	Biological outcome	Reference
MAPK signaling pathway	SCFAs	Prostate-specific PTEN KO mice	IGF1R→ERK phosphorylation↑	Activation	Prostate tumor growth↑	Matsushita et al., 2021
		MDA-MB-231 cells (breast cancer)	FFAR3→ERK phosphorylation↓	Suppression	Aggressiveness of breast cancer↓	Thirunavukkarasan et al., 2017
	DCA	HM3 colorectal cancer cells	MEK1,ERK1/2↑	ERK MAPK activation	MUC2 expression↑	Lee et al., 2010
			p38↑	p38 MAPK activation	MUC2 expression↑	Lee et al., 2010
			JNK↑	JNK MAPK activation	MUC2 expression↓	Lee et al., 2010
	KYNA	HT-29 cells	ERK1/2 and p38 phosphorylation↓	Suppression	Tumor cell proliferation↓	Walczak et al., 2014
PI3K signaling pathway	SCFAs	Prostate-specific PTEN KO mice	IGF1R→Akt phosphorylation→	Activation	Prostate tumor growth↑	Matsushita et al., 2021
	DCA	HM3 colorectal cancer cells	EGFR → PI3K → PIP^2^→PIP^3^→ Akt activation↑	Activation	MUC2 expression↑	Lee et al., 2010
	KYNA	HT-29 cells	Akt phosphorylation↓	Suppression	Tumor cell proliferation↓	Walczak et al., 2014
	Urolithin A	MiaPaCa2/BxPC3/PANC1/ K8484 PDAC cells	Akt/p70S6K phosphorylation↓	Suppression	Tumor cell proliferation↓and apoptosis↑	Totiger et al., 2019
Wnt signaling pathway	DCA	SW480/LoVo cells	β-catenin Tyr-phosphorylation↑→ uPAR/cyclin D1↑	Activation	Tumor cell proliferation↑ and invasion↑	Pai et al., 2004
	Butyrate	HCT116/SW620 cells	degradation of β-catenin by autophagy lysosome pathway	Suppression	CRC growth↑	Garavaglia et al., 2022
	KYNA	HT-29 cells	β-catenin expression↑ without nuclear translocation	–	–	Walczak et al., 2014
	Urolithin B	HepG2/Bel7402 cells (HCC)	GSK-3 β kinase Activation→β-catenin phospho (Ser33/37/Thr41) →β-catenin degradation↑ β-catenin nuclear translocation↓	Suppression	HCC proliferation↓	Lv et al., 2019
TLR4 signaling pathway	Urolithin A	BMDs	IKK activity↓	Suppression	Anti-tumor and anti-inflammatory↑	Abdelazeem et al., 2021
	Butyrate	SW480/CT26 cells	TLR4/CD14 expression↑→ERK/JNK/NF-κB p65 phosphorylation↑	Activation	Anti-tumor↑	Xiao et al., 2018

#### 2.1.1 MAPK signaling pathway

Mitogen-activated protein kinase (MAPK) is a serine/threonine protein kinase expressed in all eukaryotic cells. The MAPK signaling pathway regulates gene expression and protein translation, thereby participating in pathological and physiological processes such as cell proliferation, differentiation, apoptosis, and aging. Accumulating evidence demonstrates that diverse gut microbiota metabolites regulate tumor phenotypes by targeting core components of the MAPK pathway (e.g., kinases and transcription factors). Their regulatory modes exhibit marked variations depending on metabolite type, tumor tissue origin, and pathway microenvironment (Fang and Richardson, 2005; Guo Y.-J. et al., 2020; Stefani et al., 2021).

Short-chain fatty acids, gut microbiota metabolites primarily generated by colonic bacterial fermentation of dietary fiber, exhibit tissue-specific regulation of the MAPK pathway. In prostate-specific phosphatase and tensin homolog (PTEN) knockout mice (a prostate cancer model), SCFAs activate MAPK signaling via IGF1R-mediated ERK phosphorylation, thereby modulating prostate cancer growth (Matsushita et al., 2021). Conversely, in invasive MDA-MB-231 cells, SCFAs suppress the MAPK pathway through free fatty acid receptor 3 (FFAR3), upregulate E-cadherin expression, and reduce breast cancer invasiveness. This opposing MAPK regulation in prostate versus breast malignancies may arise from tissue-specific receptor expression profiles or tumor microenvironmental differences (Thirunavukkarasan et al., 2017).

Deoxycholic acid, a secondary bile acid, induces dose-dependent increases in p53 mRNA yet ultimately suppresses p53 protein via proteasomal degradation. In HCT116 cells, this DCA-mediated p53 inhibition partially depends on ERK signaling, revealing a novel mechanism for bile acids in colorectal carcinogenesis. Mucoprotein 2 (MUC2) is a secretory mucoprotein normally expressed by goblet cells (GC) in the intestine, which is abnormally expressed in CRC. Lee et al. (2010) found that in HM3 colorectal cancer cells, DCA not only activates the ERK and p38 MAPK pathways to promote MUC2 expression in colorectal cancer cells but also inhibits MUC2 expression through the JNK signaling pathway. This bidirectional transcriptional regulation is associated with downstream transcription factors of different pathways. Given that dysregulated MUC2 compromises intestinal barrier integrity and enhances tumor invasion, DCA’s modulation of MUC2 may influence CRC aggressiveness. Collectively, these studies implicate DCA in CRC initiation, progression, and invasion, suggesting that targeting the DCA-MAPK axis could mitigate cancer risk or suppress advancement (Lee et al., 2010).

In summary, current evidence indicates that gut microbiota metabolites primarily regulate the MAPK pathway via ERK modulation, with research predominantly focused on SCFAs and DCA. Notably, KYNA also suppresses proliferation across multiple cancer cell lines. For instance, KYNA inhibits HT-29 cell proliferation by reducing phosphorylation levels of ERK1/2 and p38 kinases (Walczak et al., 2014). Future *in vitro* studies should include more and representative cell models to enhance mechanistic robustness and translational relevance.

#### 2.1.2 PI3K signaling pathway

Phosphatidylinositol 3-kinase (PI3K) is an important kinase in phosphatidylinositol (PI), exhibiting dual activity as both a PI kinase and a serine/threonine kinase. Akt is a type of protein kinase and an important downstream signaling molecule of PI3K. In tumors, abnormal activation of the PI3K/Akt signaling pathway (such as PIK3CA mutations or PTEN loss) is one of the most common molecular events, closely associated with malignant proliferation, drug resistance, and metastatic potential of tumor cells, making it a core target pathway in cancer research (Chen et al., 2016; Alzahrani, 2019; He et al., 2021c).

Some gut microbiota metabolites regulate both the MAPK signaling pathway and the PI3K signaling pathway, such as the SCFAs, DCA, and KYNA mentioned earlier. Similar to the regulation of the MAPK signaling pathway, in Pten knockout prostate cancer models, SCFAs activate PI3K signaling via IGF1R, inducing an IGF-1 autocrine loop that synergistically promotes tumor growth (Matsushita et al., 2021). In HM3 colorectal cancer cells, DCA recruits PI3K through EGFR, catalyzing PIP^2^-to-PIP^3^ conversion to activate Akt and drive MUC2 transcription (Lee et al., 2010). Conversely, KYNA dose-dependently inhibits Akt phosphorylation in HT-29 cells, suppressing PI3K activity while may relieve transcriptional repression of p21 Waf1/Cip1, thereby inducing p21 Waf1/Cip1 overexpression and inhibiting HT-29 cell proliferation. Notably, rats tolerate intravenous KYNA (50 or 100 mg/kg/h), supporting its potential as a chemopreventive or adjuvant agent. Paradoxically, tumor-synthesized KYNA accumulates in the microenvironment while exogenous KYNA inhibits proliferation, suggesting concentration- and source-dependent effects requiring further mechanistic clarification (Walczak et al., 2014).

Beyond co-regulating MAPK and PI3K pathways in tumorigenesis, certain gut microbiota metabolites specifically target PI3K signaling. In pancreatic ductal adenocarcinoma (PDAC) models, Uro A downregulates the PI3K/AKT/mTOR pathway by inhibiting phosphorylation of Akt and p70S6K in human (MiaPaCa2, BxPC3, PANC1) and murine (K8484) PDAC cells, reducing proliferation while enhancing apoptosis. Moreover, Uro A suppresses tumor growth in xenografts and PKT mice while improving survival. Unlike KYNA, Uro A exhibits excellent drug tolerance without significant toxicity in preclinical studies. A Phase I trial confirmed its rapid absorption, high bioavailability, and tolerability, supporting its potential for dietary intervention in PDAC patients. Clinically, Uro A outperforms gemcitabine in improving overall survival. Nevertheless, Uro A-gemcitabine combination therapy failed to extend survival, potentially due to CREB-mediated chemoresistance pathway activation, necessitating optimized dosing or co-administration with pathway inhibitors for clinical translation (Totiger et al., 2019).

In summary, gut microbiota metabolites bidirectionally regulate tumorigenesis by targeting the PI3K/Akt pathway, constituting a complex network of pro- and anti-tumorigenic effects. However, the clinical translation of these findings is complicated by the concentration- and source-dependent functionalities of certain metabolites. Thus, future studies must strengthen mechanistic investigations *in vivo* and advance clinical research to facilitate therapeutic applications.

#### 2.1.3 Wnt signaling pathway

The Wnt signaling pathway is a relatively conserved signaling pathway in evolution that plays a key role in embryonic development, cell proliferation, differentiation, and orientation. The typical Wnt/β-catenin pathway drives tumorigenesis via nuclear-translocated β-catenin binding to β-catenin T-cell factor/lymphoid enhancer-binding factor (TCF/LEF) complexes, activating proto-oncogenes such as cyclin D1 and c-Myc (Duchartre et al., 2016; Krishnamurthy and Kurzrock, 2018; Parsons et al., 2021).

Gut microbiota metabolites primarily target dynamic changes in β-catenin within the Wnt/β-catenin pathway, including phosphorylation, degradation, and nuclear translocation. In CRC models, the key regulatory mechanisms are as follows: (1) Phosphorylation modulation: Low-dose DCA (5 and 50 μM) enhances β-catenin tyrosine phosphorylation in SW480/LoVo cells, inducing uPA, uPAR, and cyclin D1 expression to promote proliferation and invasion (Pai et al., 2004). (2) Degradation reprogramming: Typically, ubiquitinated phosphorylated β-catenin is degraded by the proteasome to maintain the pathway in a quiescent state. However, butyrate promotes the translocation of β-catenin to autophagosomes and autophagolysosomes in HCT116 and SW620 cells, thereby inducing the autophagolysosomal pathway to replace the proteasomal proteolytic pathway (Garavaglia et al., 2022). This not only provides an explanation for butyrate’s inhibition of CRC growth but also offers new insights into the connection between the Wnt signaling pathway and autophagy. (3) Nuclear translocation blockade: KYNA (1 mM) elevates β-catenin expression in HT-29 cells without triggering nuclear translocation, implicating non-canonical Wnt signaling or post-translational regulatory mechanisms (Walczak et al., 2014).

Given that the canonical Wnt/β-catenin pathway is activated in 80% of colorectal tumors, current research on gut microbiota metabolite-mediated regulation of this pathway primarily focuses on CRC. In fact, a wider range of metabolites and tumor-related signaling pathways await further investigation. For instance, in HepG2 and Bel7402 cells, Uro B activates GSK-3β kinase to promote β-catenin phosphorylation at Ser33, Ser37, and Thr41, triggering its degradation via the ubiquitin-proteasome pathway. Simultaneously, Uro B inhibits β-catenin nuclear translocation, impairing its association with TCF/LEF transcription factors. These mechanisms collectively suppress Wnt/β-catenin signaling and inhibit hepatocellular carcinoma cell proliferation (Lv et al., 2019).

#### 2.1.4 TLR4 signaling pathway

Toll-like receptor 4 (TLR4), a crucial pattern recognition receptor, specifically recognizes LPS from Gram-negative bacteria. TLR4 forms a complex with the coreceptors myeloid differentiation protein-2 (MD2) and CD14. In serum, LPS is transported by LPS-binding protein (LBP) to membrane-bound CD14. The resulting LPS-CD14 complex then binds to TLR4/MD2, inducing TLR4 dimerization and subsequent signal transduction. Through its intracellular TIR domain, TLR4 recruits the adaptor protein myeloid differentiation primary response 88 (MyD88), activating the canonical MyD88-dependent pathway. This leads to activation of the IKK complex and MAPK pathways (including JNK, p38 and ERK), ultimately inducing the release of proinflammatory cytokines (such as TNF-α, IL-1β, and IL-6/8). TLR4-driven inflammatory responses represent a central mechanistic link connecting diverse pathological processes including infection, tissue damage, and tumorigenesis (Gruffaz et al., 2017; Xu et al., 2017; Zhang et al., 2023).

Previous studies demonstrate that LPS upregulates vascular endothelial growth factor C (VEGF-C) expression in SW480 cells through the LPS-TLR4-NF-κB/JNK signaling pathway in time- and concentration-dependent manners, thereby promoting CRC migration, invasion, lymphangiogenesis, and lymph node metastasis (Zhu et al., 2016). Conversely, Uro A suppresses CRC progression by inhibiting IKK activity, subsequently attenuating LPS-TLR4-NF-κB signaling. Notably, NF-κB serves as a pivotal molecular nexus bridging inflammation and carcinogenesis. The TLR4-NF-κB signaling axis may represent a critical mechanistic link between Inflammatory bowel disease (IBD) and CRC, suggesting novel therapeutic and preventive strategies for IBD and CRC (Abdelazeem et al., 2021).

In addition to pathogen-associated molecular patterns such as LPS, metabolites derived from the gut microbiota have also been found to regulate the TLR4 signaling pathway, but their mechanisms of action and biological effects may be fundamentally different. In SW480 and CT26 CRC cells, butyrate activates TLR4 signaling to modulate innate immune responses. Mechanistically, butyrate upregulates TLR4 and CD14 expression, induces phosphorylation of ERK, JNK, and NF-κB p65, and stimulates TNF-α, but has no effect on IL-6 secretion (Xiao et al., 2018). This suggests that butyrate activates the TLR4 signaling pathway in a manner distinct from LPS, but may exert anticancer effects through synergistic interactions with other downstream signaling pathways. In summary, the TLR4 signaling pathway is activated by different ligands in CRC, producing complex and even opposing biological effects, highlighting the complexity of its regulation.

In summary, the TLR4 signaling pathway, when activated by ligands such as LPS and gut microbiota metabolites, can regulate tumor cell proliferation, apoptosis, invasion, and metastasis through downstream signaling pathways such as NF-κB and MAPK. Therefore, the TLR4 signaling pathway plays a crucial role in tumorigenesis and tumor progression. However, compared to other signaling pathways, there is currently a lack of research on how gut microbiota metabolites influence tumorigenesis and tumor progression by regulating the TLR4 signaling pathway, with studies primarily focusing on butyrate and Uro A metabolites in colorectal tumors.

Collectively, gut microbiota metabolites target core signaling pathways (MAPK, PI3K/AKT, Wnt/β-catenin, TLR4) to regulate tumorigenesis through two fundamental characteristics. (1) Multipathway regulation: For example, SCFAs concurrently activate both MAPK and PI3K pathways in prostate cancer models; DCA bidirectionally modulates MAPK/PI3K/Wnt signaling in CRC; KYNA suppresses MAPK/PI3K activity while exerting complex regulation on β-catenin expression in HT-29 cells. (2) Tumor-type specificity: For example, SCFAs promote tumorigenesis in prostate cancer via the MAPK pathway but inhibit tumorigenesis in breast cancer.

Notably, current evidence elucidates the significance of the gut microbiota metabolite-signaling axis in oncogenesis and reveals its therapeutic potential. Accumulating studies demonstrate that beyond modulating tumor progression, the activity of key signaling pathways critically influences radiotherapy response. For instance, PI3K/Akt pathway activation has been identified as a pivotal mediator of radioresistance in small cell lung cancer (SCLC; Jin et al., 2022), while dual PI3K/mTOR inhibition has emerged as an effective strategy to enhance radiosensitivity in breast cancer (Gasimli et al., 2023). These findings suggest that targeting and regulating signaling pathways to improve radiotherapy efficacy and mitigate its adverse effects could be a potential synergistic strategy. Given their regulatory effects on these pathways, gut microbiota metabolites represent promising candidates for novel anticancer therapies, particularly as radiosensitizers or radioprotectors.

### 2.2 Control of the tumor immune microenvironment

**FIGURE 2:**
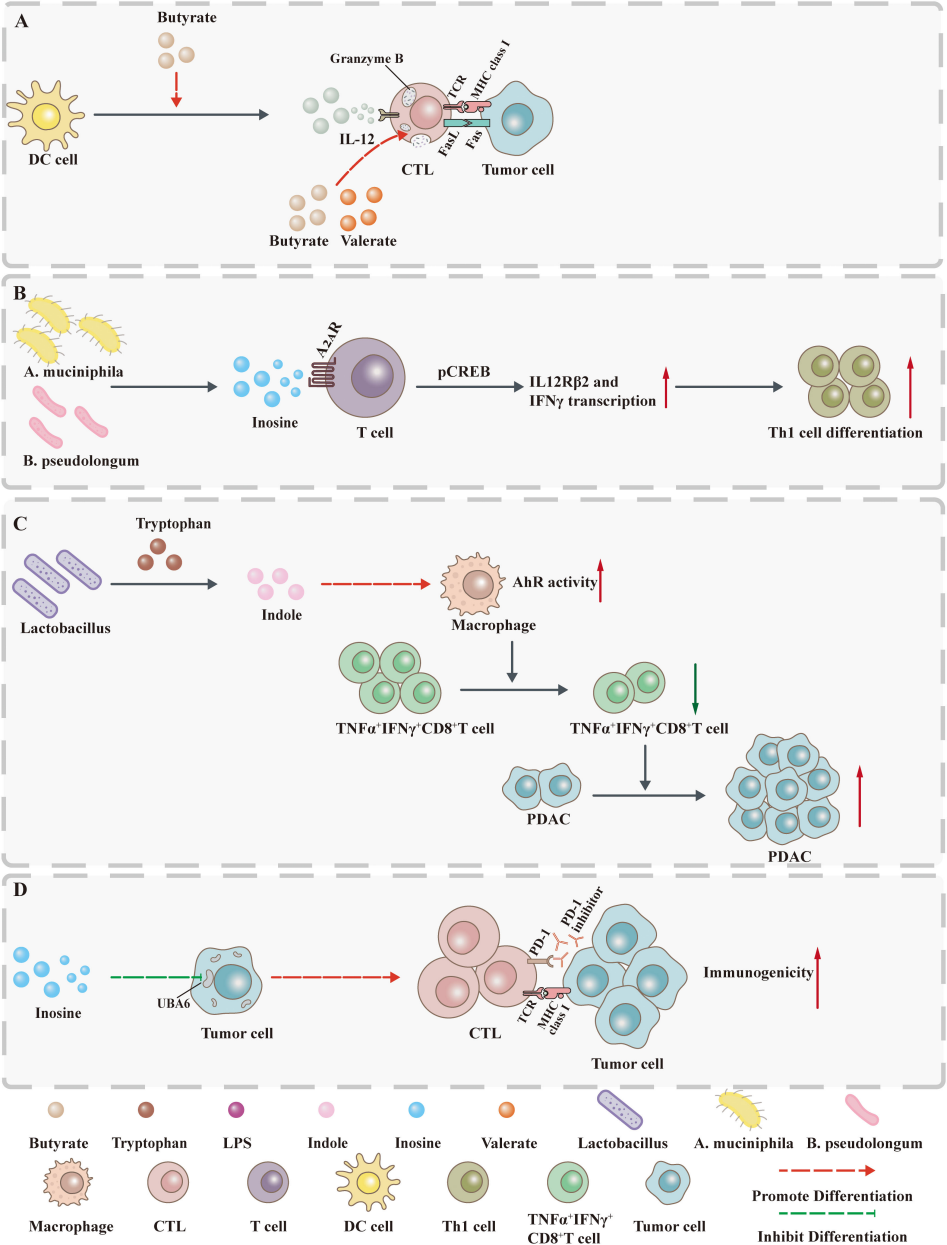
Gut microbiota metabolites influence tumor development by controlling immunity. **(A)** Butyrate not only promotes GzmB production through ID2-dependent IL-12 signaling but also directly induces GzmB expression. **(B)** Inosine acts on A_2A_R on T lymphocytes to stimulate phosphorylation of cAMP response element binding protein (pCREB) through the inosine-A_2A_R-cAMP-PKA signaling pathway, up-regulate IL12Rβ2 and IFNγ transcription, and promote Th1 cell differentiation and accumulation in the TME. **(C)** Indole increases AhR activity in tumor-associated macrophages (TAMs) and inhibits the accumulation of TNFα^+^IFNγ^+^CD8^+^ T cells within the tumor, thereby promoting the growth of PDAC. **(D)** Inosine not only enhances the sensitivity of tumor cells to T cell-mediated cytotoxicity by inhibiting the ubiquitin-activating enzyme UBA6 but also directly enhances the ability of tumor cells to present tumor antigens.

The tumor microenvironment (TME) is comprised of a variety of components, including multiple immune cell types, cancer-associated fibroblasts, tumor cells and cytokines. The significance of these components lies in their capacity to influence immune responses through complex interactions. Whether these interactions produce anti-tumor or pro-tumor effects, they play a key role in tumor immunity (de Visser and Joyce, 2023). Gut microbiota metabolites are defined as small molecules capable of diffuse transmission from the gut to other sites, with the potential to accumulate within the TME and engage with diverse components of the TME. This interaction influences both local and systemic anti-tumor immune responses (Figure 2).

#### 2.2.1 T cell

##### 2.2.1.1 Cytotoxic T cells

Cytotoxic T lymphocytes (CTLs) represent the principal effector population responsible for eliminating tumor cells expressing MHC class I molecules. Through T cell receptor (TCR) recognition of tumor antigen-MHC-I complexes and CD8 coreceptor engagement, CTLs release cytotoxic molecules including granzymes and perforin, directly inducing target cell apoptosis (Oh and Fong, 2021; Park et al., 2023).

Emerging evidence indicates that SCFAs modulate antitumor immunity through T-cell regulation. In terms of CTL cytotoxicity, butyrate exerts concentration-dependent effects on CD8^+^ T cell cytotoxicity: high concentrations (5 mM) induce apoptosis without affecting proliferation, whereas low doses significantly enhance IFN-γ and granzyme B (GzmB) production via ID2-dependent IL-12 signaling pathway activation. This suggests dose-specific immunomodulatory effects of butyrate in TME, though precise mechanisms require further elucidation. In EG7 tumor-bearing mice, *in vitro* butyrate-pretreated OT-I CD8^+^ T cells demonstrated enhanced antitumor activity, characterized by improved tumor infiltration and increased IFN-γ secretion. This effect was also validated in healthy human-derived CD8^+^ T cells (He et al., 2021b). Similarly, butyrate and valerate promote IFN-γ and GzmB production through class I HDAC inhibition and mTOR activation (independent of GPR41/43). In the CD45.2^+^ mouse B16OVA melanoma model and Panc OVA pancreatic cancer model, antigen-specific CTLs pretreated with valeric acid or butyric acid exhibited enhanced IFN-γ and TNF-α production as well as tumor clearance capacity, indicating the potential application of SCFAs in adoptive CTL therapy (Luu et al., 2021). In summary, SCFAs (especially butyrate and propionate) enhance CD8^+^ T cell function through multiple mechanisms. Their potential application in enhancing tumor immunotherapy (including adoptive CTL therapy and chemotherapy combined with immunotherapy) has been preliminarily validated. However, the effects of dose-response relationships and individual microbiota differences on their functions require further exploration to provide a theoretical foundation for clinical translation.

In terms of CTL cell production, desaminotyrosine (DAT) enhances CTL responses by directly promoting activated CD8^+^ T cell generation in combination immunotherapy. Mechanistically, *in vitro* experiments show that DAT treatment can significantly increase the proportion of CD8^+^ T cells expressing the pro-inflammatory cytokine IFN-γ only under conditions where TCR stimulation and co-stimulatory signals are present. When co-administered with anti-CTLA-4 therapy *in vivo*, DAT amplifies IFN-γ-producing CD8^+^ T cell populations and enhances natural killer (NK) cell activation. These findings establish DAT’s context-dependent immunoregulation: requiring TCR/costimulatory signals *in vitro* and immune checkpoint blockade (ICB) *in vivo*. Although oral DAT supplementation alters murine gut microbiota composition, the functional correlation between DAT-induced microbial shifts and antitumor immunity requires further validation. DAT effectively reverses the adverse effects of broad-spectrum antibiotic-induced dysbiosis on CTLA-4-mediated antitumor immunity, providing a new strategic direction for overcoming immune therapy resistance (Joachim et al., 2023). IPA, a tryptophan-derived metabolite, enhances ICB efficacy by promoting precursor exhausted T cells (Tpex) through epigenetic regulation of T cell stemness. Specifically, IPA increases H3K27 acetylation at Tcf7 super-enhancer regions to sustain CD8^+^ T cell progenitor programs (Jia et al., 2024). In summary, DAT and IPA potentiate antitumor immunity through microenvironment-dependent effector T cell activation and epigenetic maintenance of T cell stemness, respectively. This discovery may provide new drug adjuvants for personalized cancer immunotherapy and new directions for developing microbiota metabolite combination therapies.

In fact, the regulatory mechanisms of more metabolites and other immune components remain to be explored. For instance, elevated metabolic demands of tumor cells deplete nutrients and generate immunosuppressive metabolites, compromising CD8^+^ T cell function (Renner et al., 2019). Under these conditions, inosine sustains CD8^+^ T cell growth and functionality via non-glycolytic pathways *in vitro*, counteracting metabolic constraints (Wang et al., 2020). This mechanism offers new insights into enhancing the efficacy of ICB and adoptive T cell therapy in solid tumors with inosine metabolic defects. Future studies should explore methods to locally increase inosine accumulation in TME to selectively regulate T cell metabolism and enhance antitumor immune responses.

##### 2.2.1.2 Helper T cells

Helper T (Th) cells, a subset of CD4^+^ T lymphocytes, recognize antigenic peptides presented by MHC class II molecules on antigen-presenting cells. Beyond orchestrating antitumor immunity through cytokine secretion, Th cells exhibit direct tumoricidal activity and establish immunological memory, underscoring their important roles in tumor immunology (Accolla et al., 2014; Ryba-Stanisławowska, 2024).

In the regulation of Th cell differentiation, inosine exerts bidirectional regulation on Th1 differentiation through the adenosine A2A receptor (A_2*A*_R), and its effects are environment-dependent: In the presence of exogenous IFN-γ and IL-12 secreted by dendritic cells, guanosine promotes Th1 differentiation by binding to the A_2*A*_R on the surface of T cells, and significantly enhances the anticancer activity of Th1 cells in various tumors such as melanoma, bladder cancer, and CRC (He et al., 2017). Further studies showed that *in vitro*, co-stimulation with CD3/CD28 or exogenous IFN-γ also promotes Th1 differentiation, and this effect can be reversed by an A_2*A*_R antagonist (Kroemer and Zitvogel, 2020). Notably, in the absence of exogenous IFN-γ, inosine via A_2*A*_R instead inhibits Th1 and Th2 cell differentiation (Mager et al., 2020). Additionally, *A. muciniphila*, which is associated with human ICB treatment responsiveness, has been shown to utilize the inosine-A_2*A*_R signaling pathway to enhance Th1 differentiation, thereby exerting an ICB-promoting effect. This suggests that A_2*A*_R signaling may represent a complete antitumor pathway in bacterial-ICB combination therapy.

In addition, SCFAs can promote the differentiation of T cells into effector T cells and regulatory T cells. The mechanism may be related to the inhibition of histone deacetylases (HDACs) in T cells and the regulation of the mTOR pathway. However, whether their immunomodulatory effects promote antitumor immunity depends on the immune environment (Park et al., 2015). 3-oxo LCA inhibits Th17 cell differentiation by directly binding to the transcription factor retinoic acid-related orphan receptor γt (RORγt).

In summary, these microbial metabolites target signaling pathways such as A_2*A*_R and RORγt, regulate epigenetics and metabolism, and specifically regulate Th cell differentiation in different immune environments. This provides a theoretical basis for understanding the regulatory mechanisms of microbiota metabolites on tumor immunity and developing combined treatment strategies.

#### 2.2.2 Macrophages

Macrophages are the first line of defense against foreign invaders, performing functions such as phagocytosis of pathogens, antigen presentation, immune regulation, and inhibition of tumor growth and metastasis. They play a central role in both anti-infective and anti-tumor immunity. TAMs are a distinct subpopulation of macrophages that infiltrate TME and exhibit unique phenotypic and transcriptional characteristics. Under the influence of immunosuppressive factors in the TME (such as PGE-2 and IL-10), TAMs lose their ability to present antigens and directly kill tumor cells, fail to effectively activate T/NK cells, and thereby promote tumor progression. Functionally, TAMs typically exhibit a pro-tumor M2-like phenotype characterized by anti-inflammatory and immunosuppressive properties, rather than the anti-tumor M1-like phenotype characterized by pro-inflammatory and immune-promoting properties (Shapouri-Moghaddam et al., 2018; Mehta et al., 2021).

The process by which macrophages differentiate into M1 and M2 types is referred to as polarization. In terms of macrophage polarization, studies have shown that antibiotic-induced depletion of SCFAs promotes the production of M1 macrophages, leading to excessive production of pro-inflammatory cytokines and resulting in persistent intestinal immune dysfunction (Scott et al., 2018). However, in Apc^*min*/+^ mice, orthotopic xenografts, and AOM/DDS-induced CRC models, *Clostridium butyricum* (*C. butyricum*) can increase CTL infiltration and inhibit TAM and M1 TAM infiltration, thereby suppressing tumor growth (Xie et al., 2025). The mechanism by which *Clostridium butyricum* influences macrophages is associated with its binding to the GRP78 receptor on tumor cell surfaces via the surface protein secD, thereby blocking the PI3K-Akt-NF-κB signaling axis and reducing IL-6 secretion by tumor cells. Although TAM M2 polarization is an additional target of *Clostridium butyricum*, whether the butyrate it produces participates in this process requires further clarification.

In terms of macrophage production of pro-inflammatory factors, butyrate inhibits macrophage inflammatory responses by suppressing HDAC and reduces the production of pro-inflammatory mediators such as nitric oxide, IL-6, and IL-12 (Chang et al., 2014). While this suggests that butyrate may play a role in maintaining intestinal immune homeostasis, its role in tumor immunity requires further validation.

In terms of macrophage activity, in PDAC, the aryl hydrocarbon receptor (AhR) activity of TAMs is dependent on indole. Dietary intervention to reduce indole production significantly decreases the AhR signaling activity of TAMs. This not only enhances the efficacy of ICB but also promotes the accumulation of TNFα^+^IFNγ^+^CD8^+^ T cells within tumors, thereby inhibiting PDAC growth (Hezaveh et al., 2022). It is important to note that there are species differences in AhR-ligand binding affinity between mice and humans, so caution is required when extrapolating findings from mouse models to clinical settings.

In summary, these findings suggest that various gut microbiota metabolites can influence tumor progression and the antitumor effects of other immune cells by regulating macrophage polarization and function. However, further research is needed to elucidate the regulatory network of the “microbial metabolites-macrophages-antitumor immunity” axis, providing direction for clinical applications such as dietary regulation, microbial intervention, or combined metabolite and immunotherapy to precisely regulate TAMs.

#### 2.2.3 Dendritic cells

Dendritic cells (DCs), as the most potent professional antigen-presenting cells, are widely distributed in the blood, lymphoid organs, and peripheral tissues. Within TME, infiltrating DCs are responsible for recognizing, capturing, and presenting tumor-associated antigens (TAAs) in both tumor and lymphoid tissues. This process promotes the differentiation of naive T lymphocytes into tumor antigen-specific T cells, upregulates co-stimulatory molecules, and initiates antitumor immune responses. While immature DCs exhibit heightened migratory capacity, only upon maturation do they acquire the ability to effectively activate naive T cells and orchestrate adaptive immunity (Fu and Jiang, 2018; Marciscano and Anandasabapathy, 2021).

Despite the central role of DCs in antitumor immunity, current understanding of how gut microbiota metabolites regulate DC function remains limited. For instance, DCA has been shown to impair the immunostimulatory function of DCs, promote Treg differentiation, and enhance Foxp3 expression (Campbell et al., 2020). These findings have begun to delineate an immunometabolic network connecting bile acids, DCs, and Tregs. The direct mechanism through which DCA modulates DCs may involve the farnesoid X receptor -mediated nuclear signaling pathway, although further investigation is required to fully elucidate this axis.

Furthermore, studies in the Apc^*Min*/+^ mouse model of CRC demonstrated that FFAR2 agonism reduces colon tumor burden and decreases the frequency of IL27^+^ DCs within tumors. Mechanistically, FFAR2 activation downregulates genes associated with cell proliferation, NF-κB activation, apoptosis, and TLR4/IFN-γ signaling, while also suppressing NF-κB pathway activity, thereby suppressing IL27 secretion. As SCFAs are endogenous agonists of FFAR2, they are proposed to modulate DC function via analogous pathways; however, this hypothesis requires interventional validation using SCFAs (Lavoie et al., 2020). Moreover, potential species-specific differences in FFAR2 expression patterns and DC subset functionality between mice and humans necessitate further examination in clinical specimens to confirm the translatability of these mechanisms.

In summary, as DCs play a central role in capturing tumor antigens and presenting them to T cells, their functional status directly governs the induction and potency of antitumor immune responses. Future studies should focus on deciphering the molecular pathways through which gut microbiota metabolites regulate key DC functions—including migration, antigen presentation efficiency, and energy metabolism, which will advance the translational development of microbiota-based interventions in cancer immunotherapy.

Collectively, gut microbiota metabolites form a complex immunoregulatory network by targeting multiple immune cell types within the tumor immune microenvironment. For instance, butyrate enhances CTL cytotoxicity via IL-12 signaling and epigenetic mechanisms, while also driving M2-like TAM polarization through the PI3K/Akt/NF-κB axis and suppressing pro-inflammatory mediator production via HDAC inhibition. Beyond supporting CTL function through “non-glycolytic” metabolic pathways, inosine facilitates Th1 differentiation via A_2*A*_R signaling to potentiate ICB. Additionally, certain metabolites act directly on tumor cells: inosine increases tumor cell susceptibility to T cell-mediated cytotoxicity by inhibiting the ubiquitin-activating enzyme UBA6 (He et al., 2021a), and can enhance immunogenicity through upregulation of antigen presentation machinery (Bird, 2020). These findings underscore the role of gut microbiota metabolites as multi-target immunomodulators and provide critical mechanistic insights into microbiota–immune–tumor crosstalk.

The dynamic remodeling of the tumor immune microenvironment is a central determinant of radiotherapy efficacy, thereby expanding the potential applications of gut microbiota metabolites. Radiotherapy has been shown to modulate the TIME through a dual mechanism: it promotes CTL priming and enhances their antitumor functions, yet concurrently facilitates the acquisition of an effector phenotype in FOXP3^+^Helios^+^ Tregs, fostering an immunosuppressive feedback loop (Frijlink et al., 2024). Notably, STAT3 inhibition strategies targeting TAMs—such as CpG-STAT3ASO—can counteract radiotherapy-induced accumulation of M2-like TAMs, and reinvigorate the immune landscape via activation of (DCs) and M1 macrophages, ultimately improving radiotherapeutic sensitivity (Raudenská et al., 2024). These findings highlight the potential of gut microbiota metabolites as innovative adjuvants that may counteract radiotherapy resistance via remodeling the immunosuppressive microenvironment, thereby providing new translational avenues to improve tumor local control and overcome treatment resistance.

## 3 Relationship between gut microbiota metabolites and tumor radiotherapy

**FIGURE 3:**
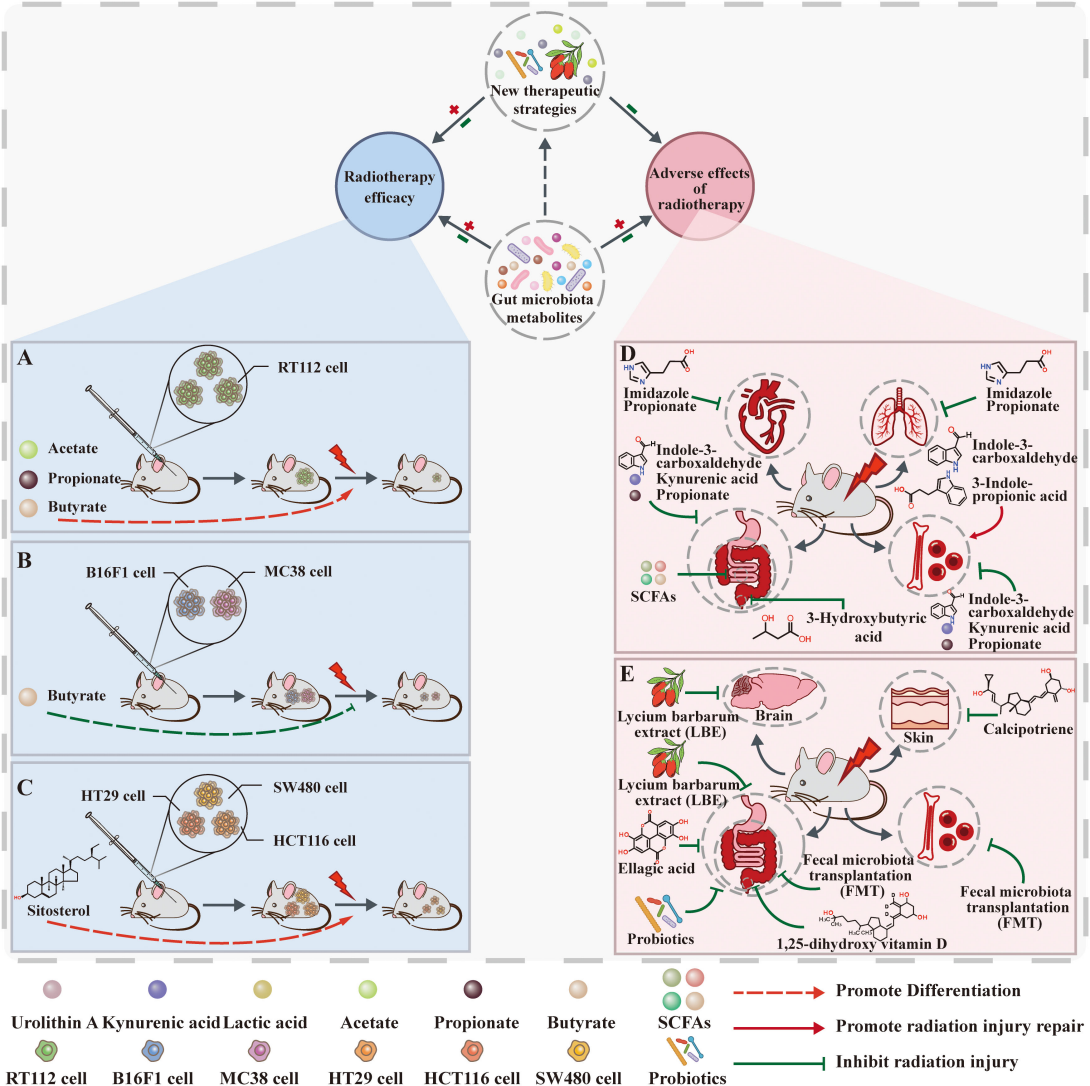
The linkage between gut microbiota metabolites and radiotherapy. **(A)** Acetate, propionate, and butyrate increase the radiosensitivity of bladder cancer cells. **(B)** Butyrate does not directly protect tumor cells from radiation but inhibits RT-induced anti-tumor immunity by suppressing STING activation. **(C)** Sterol, as a dietary supplement, may enhance the efficacy of radiotherapy and improve cancer by regulating gut microbiota and promoting apoptosis in tumor cells. **(D)** Gut microbiota metabolites influence radiotherapy’s adverse effects. Intestinal metabolites of ImP, SCFA, and inulin inhibit intestinal damage after radiation. Indole-3-acetaldehyde and 3-hydroxybutyric acid (3HB) inhibited radiation enteritis and radiation proctitis (RP), respectively. Propionic acid, indole-3-carboxaldehyde (I3A), and KYNA inhibit post-radiation hematopoietic and gastrointestinal damage. I3A and IPA promote post-radiation hematopoietic recovery. **(E)** New therapeutic strategies based on microbial metabolites affect radiotherapy’s adverse effects. Vitamin D inhibits radiation dermatitis and RP. Aqueous extract of *Lycium barbarum* inhibits radiobrain damage and radiointestinal injury. Lactic acid, urolithin A (Uro A), and probiotics inhibit radiation bowel injury. FMT inhibits radiological hematopoietic and gastrointestinal damage.

As the association between gut microbiota metabolites and tumorigenesis and tumor progression is increasingly elucidated, their potential application in tumor radiotherapy has garnered significant attention. Radiotherapy, as a key modality in tumor treatment, is administered to approximately 70% of tumor patients, with a cure rate of 40% for malignant tumors, making it an effective local treatment approach. However, while radiotherapy effectively kills tumor cells, it also tends to damage normal tissues and is associated with various side effects, which limits its clinical application. Therefore, investigating the association between gut microbiota metabolites and radiation therapy holds significant clinical importance. Current research primarily focuses on two aspects: First, the impact of radiation therapy on gut microbiota metabolites; second, the regulation of radiation therapy efficacy and adverse reactions by gut microbiota metabolites (Figure 3).

### 3.1 Radiotherapy affects the composition of the gut microbiota and its metabolites

Radiotherapy can disrupt the colonization resistance of the gut microbiota, leading to dysbiosis, with specific characteristics varying significantly depending on the radiotherapy setting and disease type. Analysis of fecal samples from cervical cancer patients undergoing radiotherapy (especially those with radiation enteritis) revealed characteristic disruptions in the gut microbiota: reduced α diversity, increased β diversity, elevated abundance of the Proteobacteria phylum, and decreased abundance of the Bacteroides genus. This suggests that the microbial balance has been disrupted, with pro-inflammatory microbiota gaining dominance, and that radiotherapy-induced dysbiosis may contribute to the onset and progression of radiation enteritis (Wang et al., 2019). In rectal cancer patients undergoing neoadjuvant radiotherapy (NART), although NART did not significantly alter gut microbiota α diversity, multiple oral pathogenic bacteria were enriched in the intestines of advanced patients, suggesting that NART may disrupt the intestinal barrier or disturb the oral-intestinal microbiota migration balance, leading to the translocation of oral pathogenic bacteria (Xu et al., 2023). These findings reveal the specific effects of radiotherapy on the gut microbiota, providing important insights into the mechanisms underlying radiotherapy-related intestinal complications.

The production of gut microbiota metabolites depends on the composition and function of the microbiota. Therefore, radiation therapy-induced disruption of the microbiota can further alter the metabolite profile. In patients with differentiated thyroid cancer (DTC), fecal microbiota structure significantly changed after ^131^I therapy (with the *Ruminococcus* family becoming the dominant genus), and levels of ARA (ARA) and linoleic acid-related metabolic pathways decreased, suggesting that 131I therapy may exacerbate radiation damage by disrupting microbiota composition and inhibiting the ARA metabolic pathway (Lu et al., 2024). In the feces of CRC patients who responded to neoadjuvant chemoradiotherapy (nCRT), butyrate-producing bacteria were enriched, and SCFA levels were significantly elevated, suggesting that gut microbiota metabolites may be closely associated with radiotherapy efficacy (Sánchez-Alcoholado et al., 2021). In a mouse model of RP and in patients undergoing radiotherapy, the abundance of *A. muciniphila* and the concentration of 3HB were significantly reduced in feces and serum, and 3HB levels were negatively correlated with the extent of radiation damage. The specific mechanism involves 3HB inhibiting the GPR43 receptor-mediated signaling pathway, thereby downregulating IL-6 expression and alleviating radiation-induced intestinal damage. This finding suggests that 3HB may be a potential radioprotective agent (Ge et al., 2024). Furthermore, the fluctuation patterns of lactic acid in the feces of mice exposed to 0, 2, 4 and 8 Gy radiation were similar between the low-dose (0, 2 Gy) and high-dose (4, 8 Gy) groups, suggesting that lactic acid may be an organic acid sensitive to radiation exposure, with its metabolic changes being associated with radiation dose. This provides a new target for exploring the effects of radiation on gut microbiota metabolism (Sakamoto et al., 2019).

In summary, radiotherapy can induce dysbiosis of the gut microbiota and alterations in the metabolite profile. These findings reveal the central role of the radiotherapy-gut microbiota-metabolite axis in radiation damage and radiotherapy efficacy, and provide important evidence for elucidating the mechanisms of radiotherapy-related complications and developing therapeutic targets.

### 3.2 Positive effects of gut microbiota metabolites on radiotherapy efficacy

Recent advances have elucidated the multifaceted mechanisms of radiotherapy: beyond directly inducing DNA damage to exert cytotoxic effects, it remodels the tumor immune microenvironment and triggers antitumor immunity (Demaria et al., 2004, 2016; Apetoh et al., 2007; Kepp et al., 2011). Current investigations into how gut microbiota metabolites modulate radiotherapy efficacy have primarily centered on SCFAs, with butyrate receiving significant attention (Table 2).

**TABLE 2:** Dual effects of butyrate in radiotherapy efficacy.

Research direction	Cell/animal model	Dose	Molecular mechanism	Reference
Tumor radio-sensitization	RT112 human bladder cancer cells	0–8 Gy	Histone acetylation ↑	Then et al., 2020
	Patient-derived CRC organoids	3 × 5 Gy	Activating the FOXO3A pathway →Ki-67^+^ cells and S-phase cells↓	Park et al., 2020
Anti-tumor immune suppression	B16-OVA tumor-bearing mice (C57BL/6J mice)	21 Gy	DCs antigen-presenting ability ↓, IFN-γ↓→tumor-specific CD8^+^ T cells↓	Uribe-Herranz et al., 2019
	MC38-OVA tumor-bearing mice (C57BL/6J mice)	20 Gy	Inhibiting STING-TBK1-IRF3 phosphorylation → Type I IFN ↓→tumor-specific CD8^+^ T cells↓	Yang et al., 2021

In local tumor control, one study in RT112 cells irradiated with 0–8 Gy demonstrated that acetate, propionate, and butyrate enhanced radiosensitivity through increased histone acetylation. In a mouse xenograft model of human bladder cancer, a high-fiber diet significantly improved tumor control after irradiation (6 Gy), which correlated with an elevated abundance of *Bacteroides acidifaciens* in the gut microbiot a (Then et al., 2020). However, the causal role of *B. acidifaciens* in radiosensitization and whether SCFAs mediate this effect remain to be established. Furthermore, given the involvement of both gut microbiota and radiation in immune regulation, future studies using immunocompetent animal models are warranted to delineate the mechanisms underlying immune-mediated radiosensitization.

In CRC research, the radiosensitizing potential of acetate, propionate, and butyrate was investigated using patient-derived 3D organoids subjected to 3 × 5 Gy irradiation. Butyrate alone enhanced radiotherapeutic efficacy, mechanistically attributed to FOXO3A-mediated reduction in Ki-67^+^ cells and S-phase arrest. Notably, a subset of FOXO3A-low organoids were non-responsive to butyrate, suggesting interindividual variation in treatment efficacy. More importantly, butyrate exerted selective protection in normal intestinal mucosa organoids: it did not exacerbate radiation-induced damage and instead promoted post-irradiation regeneration (Park et al., 2020). These findings propose butyrate as a promising dual-function agent that simultaneously radiosensitizes tumors and protects normal tissue, highlighting its potential as a “sensibilization without toxicity” strategy in CRC radiotherapy.

In terms of radiotherapy-induced antitumor immunity, vancomycin has been demonstrated to enhance radiotherapy-induced antitumor immunity and suppress tumor growth, a synergistic effect dependent on cross-presentation of tumor antigens to CD8^+^ T cells and IFN-γ signaling. However, in mice receiving 21 Gy irradiation combined with vancomycin, butyrate supplementation reduced the frequency of OVA-presenting DCs in lymph nodes and decreased tumor-specific T cell infiltration. *In vitro* assays further confirmed that butyrate impairs the antigen-presenting function of DCs and suppresses IFN-γ secretion (Uribe-Herranz et al., 2019). These findings indicate that butyrate compromises APC activity and disrupts cross-priming, thereby antagonizing vancomycin’s enhancement of radiotherapy-induced antitumor immunity. Similarly, in C57BL/6J mice bearing MC38-OVA tumors irradiated with 20 Gy, butyrate inhibited the activation of tumor-specific CD8^+^ T cells by radiation-primed DCs. Mechanistically, butyrate suppressed STING-dependent phosphorylation of TBK1 and IRF3, attenuating type I interferon production in DCs and ultimately abrogating radiation-induced cytotoxic T cell responses (Yang et al., 2021).

In summary, butyrate exhibits a “double-edged sword” effect in radiotherapy: on the one hand, it enhances histone acetylation and activates the FOXO3A pathway to block S-phase cells, thereby increasing local tumor radiosensitivity; on the other hand, it impairs the antigen-presenting function of (DCs), weakening CD8^+^ T cell activation and IFN secretion, thereby antagonizing radiotherapy-induced systemic antitumor immunity. This context-dependent duality underscores the need to evaluate its clinical utility based on treatment intent and context-specific TME features. Future studies should focus on establishing causal mechanisms and validating its effects across tumor types to clarify the functional boundaries of butyrate-mediated radio-modulation. The development of targeted delivery approaches may help circumvent immunosuppressive risks while maximizing its local radiosensitizing benefits.

### 3.3 Negative effects of gut microbiota metabolites on radiotherapy adverse effects

Radiotherapy plays an indispensable role as an important adjuvant treatment for malignant tumors in the abdominal cavity, retroperitoneum, and pelvis in clinical practice. However, its efficacy is often limited by complications such as radiation-induced intestinal injury, which exhibits dose-limiting toxicity that frequently leads to treatment interruption or dose reduction, thereby compromising tumor control and survival (Lam et al., 2019; Sassa, 2020). Identifying key factors influencing the development and severity of radiotherapy-related adverse effects is therefore essential for optimizing dosing strategies and improving therapeutic safety and efficacy (Table 3).

**TABLE 3:** Protective effects and mechanisms of gut microbiota metabolites in radiation therapy-induced damage.

Radiotherapy injury type	Gut microbiota metabolites	Dose	Cells/animal models	Molecular mechanism	Biological outcome	Reference
Radiation-induced intestinal fibrosis	Dietary pectin (precursor of SCFAs))	10 Gy single abdominal γ-ray irradiation	pVillin-Cre-EGFP double-transgenic mice	–	Ileal submucosal thickness↓; EMT↓	Yang et al., 2017
Chronic radiation-induced intestinal fibrosis	Inulin metabolites (containing SCFAs)	15 Gy abdominal irradiation	C57BL/6J mice	–	Colonic fibrosis↓	Ji et al., 2024
		10 Gy	NIH/3T3 cells	Fibrosis-related genes, collagen synthesis genes, extracellular matrix pathway gene expression↓	Colonic fibrosis↓	Ji et al., 2024
Radiation proctitis	3HB	25 Gy pelvic irradiation	C57BL/6J mice	Block GPR43→IL-6 signaling pathway↓	Intestinal epithelial injury↓	Ge et al., 2024
Radiation proctitis	Propionate	8 Gy abdominal irradiation	BALB/c mice	Activate GPR43→Histone acetylatio↑→Expression of Occludin/ZO-1/mucin↑	Intestinal barrier function↑	He et al., 2023
Radiation-induced intestinal epithelial injury	I3A	13 Gy total abdominal irradiation	C57BL/6J mice	Activate AhR/IL-10/Wnt signaling axis	Intestinal epithelial proliferation↑	Xie et al., 2024a
Hematopoietic system injury	ARA	4 Gy total body irradiation	C57BL/6J mice	TNF-α/IL-6; Oxidative stress ↓→Protect HSC niche	Thymus/spleen weight↑; Peripheral blood cell recovery↑	Lu et al., 2024a
Hematopoietic system injury	IPA	4 Gy total body irradiation	C57BL/6J mice	–	Myelosuppression↓; Recovery of hematopoietic organs and gastrointestinal tract↑	Xiao et al., 2020
Hematopoietic system injury	I3A	5 Gy total body irradiation	C57BL/6J mice	Quiescent state of HSPC↑→Radiation resistance↑ ROS ↓→Inhibit apoptosis	Hematopoietic stem and progenitor cell regeneration↑;Peripheral blood recovery↑	Guan et al., 2024
Radiation-induced Lung Injury	PGF2α	15 Gy total lung irradiation	C57BL/6J mice	–	Pulmonary inflammation↓; Respiratory function↑	Chen Z.-Y. et al., 2021
	PGF2α	6 Gy	BEAS-2B/MLE-12 cells	Activate FP/MAPK/NF-κB pathway	Lung cell proliferation↑; Lung cell apoptosis↓	Chen Z.-Y. et al., 2021
Radiation-induced cardiopulmonary Injury	L-histidine; ImP	15 Gy total thoracic irradiation	C57BL/6J mice	–	Lung function↑; Cardiac contractile function↑	Chen Z. et al., 2021
	ImP	6 Gy	BEAS-2B cells	Inhibit NF-κB→GSDMD transcription↓→Pyroptosis↓	Lung cell proliferation↑	Chen Z. et al., 2021

#### 3.3.1 Gastrointestinal adverse reactions

Radiation-induced intestinal damage is a common complication of radiotherapy for abdominal and pelvic tumors, including intestinal fibrosis and radiation enteritis, which severely affect patients’ quality of life. In recent years, the regulatory role of the intestinal microbiota and its metabolites in this type of damage has become a hot topic of research.

In radiation-induced intestinal fibrosis, EMT has been implicated as a key mechanism. In pVillin-Cre-EGFP double transgenic mice subjected to a single 10 Gy abdominal γ-irradiation, dietary pectin supplementation not only reversed radiation-induced ileal submucosal thickening but also reduced the number of cells co-expressing α-SMA/EGFP and vimentin/EGFP, indicating suppression of EMT. Although pectin significantly increased SCFA levels, whether SCFAs mediate EMT inhibition through HDAC suppression or GPCR signaling remains to be experimentally established (Yang et al., 2017). Furthermore, the use of single high-dose irradiation in mice diverges from clinical fractionation regimens, underscoring the need for more clinically relevant models to improve translational applicability.

In a murine model of chronic radiation enteropathy (15 Gy abdominal irradiation), transplantation of gut microbiota and metabolites from inulin-fed donors attenuated colonic fibrosis. *In vitro* studies further demonstrated that inulin-derived metabolites suppress the expression of pro-fibrotic genes, collagen synthesis genes, and extracellular matrix pathway genes in NIH/3T3 cells. These findings suggest a novel translational strategy for preventing chronic radiation-induced colon fibrosis. Notably, although inulin enriched SCFA-producing bacteria and elevated fecal SCFAs, the specific mechanisms through which SCFAs mitigate fibrosis remain to be elucidated (Ji et al., 2024).

In radiation-induced enteritis, reduced abundance of *Akkermansia muciniphila* and decreased levels of the microbial metabolite 3-hydroxybutyrate (3HB) were observed in both a mouse model (25 Gy irradiation) and patient samples, accompanied by elevated IL-6. Experimental studies demonstrated that 3HB attenuates intestinal epithelial damage by inhibiting the GPR43-mediated IL-6 signaling pathway. Oral supplementation with *A. muciniphila* increased 3HB levels and enhanced radioprotective effects (Ge et al., 2024). However, an alternative mechanism was reported in a separate study: following single-dose abdominal irradiation (8 Gy) in mice and abdominal radiotherapy in patients, reduced abundance of *A. muciniphila* led to decreased propionate production. Propionate enhances intestinal barrier function by activating GPR43, promoting histone acetylation, and upregulating tight junction proteins (Occludin and Zonula occluden-1 (ZO-1)) and mucin expression. The opposing effects of 3HB and propionate on GPR43 signaling suggest ligand-specific regulatory complexity that warrants further investigation (He et al., 2023).

In cervical cancer patients who developed enteritis after pelvic radiotherapy, levels of indole-3-acetaldehyde were significantly reduced. FMT from healthy donors mitigated intestinal epithelial damage in 9 Gy-irradiated C57BL/6J mice and restored indole-3-acetaldehyde levels, possibly through attenuation of radiation-triggered immune inflammation (Tu et al., 2024). Although the precise mechanism of indole-3-acetaldehyde-mediated protection remains unclear, these findings support the therapeutic potential of FMT and highlight indole-3-acetaldehyde—a tryptophan metabolite—as a promising target for preventing or treating radiation-induced enteritis.

In promoting intestinal epithelial repair, oral administration of I3A in a C57BL/6J mouse model of radiation-induced enteropathy (13 Gy whole-abdomen irradiation) stimulated intestinal epithelial proliferation and improved survival through activation of the AhR/IL-10/Wnt signaling axis, suggesting its potential as a therapeutic target for radiation-induced intestinal injury (Xie et al., 2024a).

In summary, gut microbiota metabolites confer multi-mechanistic protection against radiation-induced intestinal damage by suppressing EMT-driven fibrosis, inhibiting the GPR43/IL-6 inflammatory pathway, and activating the AhR/Wnt-mediated epithelial repair program. However, the precise molecular mechanisms, safety profiles, and efficacy of these metabolites must be thoroughly investigated to facilitate the clinical translation of precision radioprotective strategies.

#### 3.3.2 Hematopoietic system injury

Ionizing radiation is highly sensitive to the hematopoietic system, causing atrophy of hematopoietic organs such as the thymus and spleen, reduction of peripheral blood cells, and disruption of the hematopoietic stem cell (HSC) microenvironment. Its protection and repair have always been a research focus in the field of radiation medicine. In recent years, the role of gut microbiota metabolites in promoting hematopoietic recovery after radiation has gradually attracted attention.

In terms of promoting the recovery of hematopoietic organs, administration of ARA to C57BL/6J mice following 4 Gy irradiation restored thymic and splenic mass and countered radiation-induced peripheral cytopenia (Lu et al., 2024a). Mechanistically, ARA acts by suppressing pro-inflammatory cytokines (e.g., TNF-α, IL-6) and oxidative stress, thereby preserving the hematopoietic stem cell niche, though its precise molecular targets remain unidentified. Separately, in mice subjected to 4 Gy whole-body irradiation, oral IPA supplementation attenuated systemic inflammation, mitigated myelosuppression, and enhanced the recovery of both hematopoietic and gastrointestinal tissues. Although the pregnane X receptor (PXR)/acyl-CoA-binding protein (ACBP) signaling pathway has been implicated in IPA-mediated radioprotection, it remains unclear whether this axis directly underlies hematopoietic recovery (Xiao et al., 2020).

In terms of promoting the regeneration of hematopoietic stem and progenitor cells (HSPCs), I3A treatment in C57BL/6J mice mice (5 Gy irradiation) not only accelerates the recovery of peripheral blood cells but also promotes the regeneration of HSPCs. Mechanistically, I3A enhanced radioresistance by inducing HSPC quiescence and reduced apoptosis through suppression of reactive oxygen species (ROS) generation. These findings identify I3A as a potential therapeutic agent for ionizing radiation-induced bone marrow suppression (Guan et al., 2024).

Furthermore, ursodeoxycholic acid (UDCA) has been shown to inhibit the FXR receptor, thereby enhancing NF-κB-dependent hematopoietic recovery, offering a promising therapeutic strategy for radiation-associated hematopoietic recovery (RAHR; Jiao et al., 2025).

In summary, gut microbiota metabolites contribute to the restoration of hematopoietic organs by mitigating inflammation and oxidative stress, modulating key signaling pathways, and facilitating HSPC regeneration, thus helping to maintain the hematopoietic stem cell niche. While these findings provide valuable insights into interventions for radiation-induced hematopoietic injury, further studies are essential to elucidate the detailed mechanisms and address pivotal challenges in clinical translation.

#### 3.3.3 Radiation-induced cardiopulmonary injury

Radiation-induced cardiopulmonary injury is the most common refractory complication of thoracic tumor radiotherapy. Despite continuous innovations in precision radiotherapy techniques, 30% of patients undergoing thoracic radiotherapy still develop radiation-induced lung injury (RILI), and there are currently no safe and effective preventive or therapeutic measures available. Recent studies have confirmed the important role of the gut-lung axis, with gut microbiota metabolites able to act on distant cardiopulmonary tissues via the bloodstream, providing an important perspective for the development of new therapies.

In terms of radiation-induced lung injury, oral administration of PGF2α significantly alleviates pulmonary inflammatory infiltration and improves respiratory function in C57BL/6J mice subjected to 15 Gy whole-lung irradiation. Its mechanism of action was validated in BEAS-2B and MLE-12 cells (6 Gy irradiation) through activation of the FP/MAPK/NF-κB pathway, thereby promoting lung cell proliferation and inhibiting apoptosis. Additionally, silencing MAPK weakened the protective effect of PGF2α on irradiated lung cells. This suggests that PGF2α could serve as a potential radioprotective agent against radiation-induced lung injury (Chen Z.-Y. et al., 2021).

In terms of radiation-induced cardiovascular injury, oral supplementation with L-histidine improved pulmonary and cardiac function in C57BL/6J mice following 15 Gy whole-thorax irradiation. Its downstream metabolite, imidazole propionate (ImP), similarly attenuated radiation-induced cardiopulmonary toxicity. In BEAS-2B cells exposed to 6 Gy irradiation, ImP was shown to suppress NF-κB activation, inhibit GSDMD transcription, reduce pyroptosis, and promote epithelial proliferation. Notably, antibiotic-mediated microbiota ablation abolished systemic absorption of orally administered ImP, indicating that its radioprotective effects are strictly microbiota-dependent. Future studies should focus on identifying the bacterial taxa responsible for ImP synthesis and delineating the mechanisms by which Gram-positive bacteria facilitate its absorption (Chen Z. et al., 2021).

In summary, gut microbiota metabolites have been demonstrated to alleviate radiation-induced cardiopulmonary injury through mechanisms including modulation of the FP/MAPK/NF-κB pathway and suppression of pyroptosis, via the gut–lung and gut–heart axes, highlighting their potential as radioprotective agents. However, current evidence remains limited, and future studies should expand the scope to include more microbiota-derived metabolites and incorporate clinical validation to strengthen the mechanistic and translational foundations for preventing and treating radiation-induced cardiopulmonary injury.

Collectively, a complex bidirectional regulatory relationship exists between gut microbiota metabolites and tumor radiotherapy: irradiation perturbs the microbiota’s structure and function, thereby altering metabolite profiles, while metabolites in turn modulate key processes including tumor radiosensitivity, immune responses, and tissue repair pathways, collectively influencing both the efficacy and toxicity of radiotherapy. For clinical translation, it is essential to not only develop more representative animal models to strengthen the preclinical evidence but also to address the discrepancy between single high-dose irradiation commonly used in animal studies and the fractionated regimens applied clinically, which differ substantially in biological effect. Thus, additional clinical trials are warranted to validate these mechanisms and therapeutic strategies in humans.

It is worth noting that host factors (genetic factors, lifestyle, living environment, etc.) can influence the composition of the host’s gut microbiota and metabolites, thereby affecting the efficacy and adverse reactions of radiotherapy. For instance, FMT from sex-matched donors enhanced survival in irradiated animals, increased peripheral leukocyte counts, and improved gastrointestinal function and epithelial integrity (Cui et al., 2017). Oral/gut dysbiosis driven by dietary or lifestyle factors may promote chronic inflammation, immune dysregulation, and metabolic dysfunction, thereby facilitating tumorigenesis, progression, and therapy resistance in head and neck cancer (Raudenská et al., 2024). These findings highlight the multidimensional influence of host factors on the microbiota–immune–metabolism axis in shaping radiotherapeutic outcomes. Further research is needed to elucidate how host-dependent microbial and metabolic variations influence radiotherapy response, thereby informing personalized treatment strategies.

## 4 New applications of microbiota-derived metabolites in radiotherapy

Cancer microbiota in clinical trials is based on the molecular mechanisms that influence cancer development, and the number of ongoing or completed clinical trials aimed at enabling microbial therapies continues to grow. In Banna et al. (2017) found that the use of *Lactobacillus rhamnosus* prevented the development of diarrhea in patients undergoing radiotherapy (Banna et al., 2017). In Cui et al. (2017) reported that fecal flora transplantation could be a therapeutic tool to improve the prognosis of patients following radiotherapy (Cui et al., 2017). We summarize several innovative therapeutic strategies based on microbial metabolites that can inhibit cancer progression, mitigate the adverse effects of radiotherapy and enhance its efficacy (Figure 3). These strategies are listed in Table 4.

**TABLE 4:** Exploring new strategies for cancer radiotherapy based on an understanding of metabolites of microbial origin.

New applications	Intestinal metabolites	Radiotherapy	Type of cancer	Toxicity type	Mechanism	Outcome	Reference
Steroidal alcohol	L. pentosan metabolites, SCFAs	–	CRC	*In vivo*	Decreased PI 3 K/Akt activity	Maintains the intestinal microbiological environment and enriches beneficial bacteria	Ma et al., 2019
LBE	SCFA and lactic acid producing bacteria	Single-dose Gy TBI	–	*in vivo* and *in vitro*	Activation of the immune response and regulation of gut microbiota and radiation-associated metabolites	Regulation of levels of specific metabolites beneficial to host function	Zheng et al., 2021
FMT	SCFAs, n-3PUFA, etc.	Mice: 9 Gy; patients: 45–50.4 Gy	cervix	*in vivo*	Restoration of beneficial flora, regulation of tryptophan metabolites, etc.	Recovery of *Trichoderma reesei* and specific downstream tryptophan metabolites	Tu et al., 2024
Urolithin A (Uro A)	–	9 Gy	–	*in vivo*	Inhibition of p53-p53-mediated apoptosis and remodeling of gut microbes	Improved maintenance of homeostasis in the gut and regeneration from radiation exposure	Zhang et al., 2021
Probiotics	SCFA, indole derivatives and other gut microbiota metabolites	13 Gy	–	*in vivo*	Improvement of inflammatory symptoms and regulation of oxidative stress	Mixed probiotics and their metabolites may promote gut recovery	Xie et al., 2024b
–	Microbiota-derived I3A	13 Gy	CRC	*in vitro* and *in vivo*	Activates AhR/IL-10/Wnt signaling pathway and upregulates probiotic abundance	I3A protects the gut from radiation damage	Xie et al., 2024a
Vancomycin	–	21 Gy	A murine melanoma tumor model	*in vivo* and *in vitro*	Increased number and enhanced function of tumor cytolytic CD 8^+^ T cells within tumours	Enhancement of RT-induced anti-tumor immune response	Uribe-Herranz et al., 2019
–	SCFAs, I3 A and KYNA	Whole body: 8 Gy; localized tumor: 10 Gy	melanoma	*in vivo*	Reduces levels of pro-inflammatory cytokines	Protection of hematopoietic and gastrointestinal systems	Guo H. et al., 2020
–	Gut microbial-derived L-histidine and its secondary metabolite ImP	Mice: 15 Gy; Cells: 4 Gy or 6 Gy	–	*in vivo* and *in vitro*	Inhibition of NF-κB expression after radiation exposure	L-histidine or ImP counteract radiation therapy-induced cardiopulmonary injury	Chen Z. et al., 2021
–	PGF 2 α	Mice: 15 Gy; Cells: 6 Gy	–	*in vivo* and *in vitro*	Inhibition of lung cell apoptosis through activation of the FP/MAPK/NF-κB axis	Promotes cell proliferation and inhibits apoptosis	Chen Z.-Y. et al., 2021

### 4.1 Dietary supplements

Enhancement of anticancer therapy can be achieved through immunomodulation and/or secretion of metabolites, including butyrate (He et al., 2021b; Luu et al., 2021), inosine (Mager et al., 2020), and Trimethylamine-N-oxide (TMAO; Wang et al., 2022). Dietary fiber is fermented by gut microbiota to produce SCFA and a wide range of other metabolites (Eaton et al., 2022). For cancer patients undergoing radiotherapy, supplementation with several different types of fiber may help reduce the side effects of radiotherapy. For example, psyllium is effective in reducing the incidence and severity of radiation-induced diarrhea (Murphy et al., 2000). When it comes to controlling tumors, the combination of psyllium and inulin showed the most significant impact. Inulin was found to slow down tumor growth in mice with breast cancer (Taper and Roberfroid, 1999) and improve tumor control and radiation response in mice with bladder tumors (Then et al., 2020). This highlights inulin’s anti-tumor properties in various cancers and its ability to boost the effectiveness of cancer treatments.

Sterol is a sweet potato extract that is widely recognized as a safe and effective natural nutritional supplement. The anticancer activity of components from natural plant or food sources has been widely reported. Sterol was found to induce tumor apoptosis by promoting the production of SCFAs by gut microbiota. Analysis of 16SrDNA revealed a significant decrease in microbiota diversity, especially in the anaplastic bacilli and thick-walled bacilli phylum, within the intestinal tracts of mice with tumors. However, administering sterol treatment restored these alterations (Ma et al., 2019), preserved a diverse microbial ecosystem, and led to the generation of beneficial metabolites, including an increase in SCFAs. SCFAs can lower the phosphorylation of PI3K and Akt at the Ser473 site in tumor tissues. This results in a reduction of the Bcl-2 associated death promoter (Bad), a decrease in the expression of the Bad-regulated mitochondrial protein Bcl-xl, and an increased release of mouse cytochrome C. The release of cytochrome C from the mitochondrial membrane space into the cytosol is a significant event in caspase-dependent apoptosis in tumor cells (Lu et al., 2016). As a result, elevated levels of caspase-9 and caspase-3 in mice treated with steroids promoted the cleavage of the DNA repair enzyme poly ADP-ribose polymerase (PARP), resulting in the apoptosis of tumor cells. Additionally, L. pentosan is significantly enriched with sterols in the gut, which is advantageous and demonstrates a strong resilience to the acidic conditions of the gastrointestinal tract (Bendali et al., 2017; Sun et al., 2017). Therefore, it is possible to use sterol as a dietary supplement to enhance the gut microbiota to ameliorate cancers, especially those of the digestive tract.

Additionally, interest is mounting regarding the potential of vitamin D to help prevent side effects linked to radiation therapy. A study by Mukai et al. (2018) showed that vitamin D supplementation was an important factor in prolonging metastasis-free survival after preoperative radiotherapy in patients with (PDAC). Radiation dermatitis is a common side effect of radiation therapy in cancer patients, and the use of vitamin D ointment can help prevent this condition (Nasser et al., 2017). A case report indicated that administering vitamin D supplements before surgery and radiation therapy in patients with recurrent breast cancer changed specific biological cancer markers, including the estrogen receptor, human epidermal growth factor receptor (EGFR), and the nuclear protein Ki67. Vitamin D deficiency is associated with the severity of RP in cancer patients (Ghorbanzadeh-Moghaddam et al., 2015). 25(OH)D3 serves as the pharmacologically active form of vitamin D. Moreover, high - dose vitamin D3 supplementation exerts beneficial effects on the human gut microbiota, as it notably reduces typical opportunistic pathogens and increases the abundance of bacterial types (Bashir et al., 2016). However, high-dose supplementation may carry risks such as hypercalcemia, and the specific dosage must be strictly controlled. Butyrate, a by-product of the microbial breakdown of carbohydrates, has a well-defined role in preventing mucosal inflammation. Sun et al. (2017) showed that reduced expression of vitamin D receptors in the intestinal epithelium led to reduced butyrate production and intestinal barrier inflammation (Kanhere et al., 2018). The ways in which vitamin D alleviates the side effects of radiotherapy could be investigated further to create suitable management guidelines and recommendations for patients receiving radiotherapy.

### 4.2 Probiotics

Prebiotics are organic compounds that the host does not digest or absorb, yet they can specifically enhance the growth and activity of beneficial bacteria in the body, leading to improved health for the host. Goji berry is a popular traditional Chinese herb and tonic recognized for its various biological effects, such as enhancing the immune system, providing antioxidant benefits, reducing inflammation, combating tumors, and protecting the kidneys and liver. Recently, goji berries have attracted attention as a potential prebiotic. It was found that *Lycium barbarum* extract (LBE) altered the bacterial composition of irradiated mice by increasing the relative abundance of SCFA and lactic acid-producing bacteria (Zheng et al., 2021). SCFA acted as agonists along with G-protein coupled receptors expressed by intestinal epithelial cells (IEC) and immune cells (Kim et al., 2013; Koh et al., 2016), modulated Treg cell homeostasis, improved epithelial barrier function, and down-regulated the pro-inflammatory responses (Smith et al., 2013; Kelly et al., 2015; Rooks and Garrett, 2016). Lactate has been shown to protect mice from chemotherapy and radiation-induced intestinal damage (Lee et al., 2018). Also, lactate serves as a substrate for the production of propionate in the acrylate pathway (Reichardt et al., 2014), producing beneficial immunomodulatory effects. LBE treatment enhanced several metabolic pathways in the gut microbiota, leading to an increase in tryptophan metabolism, arginine and ornithine metabolism, indole alkaloid production, and secondary bile acid synthesis. It also raised the levels of dopamine and L-acetyl glycine in tyrosine metabolism, as well as tetrahydrofolate (THF) in folate biosynthesis. Additionally, there was a reduction in bacteria associated with ARA metabolism, along with decreased levels of ARA and other organic heterocyclic compounds like methyl pyrazine and 4-pyridoxic acid (Zheng et al., 2021). Indole derivatives enhance barrier function and increase the expression of IL-1β in epithelial cells, while also aiding in the preservation of type 3 innate lymphocytes. These lymphocytes are crucial for antimicrobial defense, tissue damage protection and repair, as well as the acute phase response (Reichardt et al., 2014). Indole metabolites promote the expression of IL-10 receptors on IEC through a mechanism that relies on the AhR (Alexeev et al., 2018), and prevent drug-induced enteropathy by reducing neutrophil infiltration (Whitfield-Cargile et al., 2016). N-Ornitho-L-taurine is another metabolite that is notably increased by LBE. Taurine is known to have various crucial functions in the body, such as interacting with bile acids, acting as an antioxidant, maintaining calcium balance, aiding in detoxification, and modulating nerve activity. Additionally, it has been demonstrated to reduce brain damage caused by gamma radiation in male rats in a time-dependent fashion (El-Maraghi et al., 2018), and to enhance the epithelial barrier function (Levy et al., 2015) by promoting IL-18 production by epithelial cells. IEC are particularly vulnerable to ionizing radiation, but LBE enhances the survival of irradiated IEC-6 cells and offers protection against radiation-induced intestinal damage. Our findings indicate that LBE supplementation raised the levels of serum interleukin-6 (IL-6) and IL-1β, both of which play a role in preventing and treating radiation injuries. LBE also down-regulated neutrophils and macrophages and attenuated the early onset acute inflammatory response after irradiation. This study confirms the radio-protective efficacy of LBE, suggesting that L. citriodora may be an immune activator and a potential prebiotic to attenuate radiation damage. However, the study also has obvious limitations. In survival experiments, C57BL/6J mice treated with 9.0 g/kg LBE fared better, while the effect of LBE was weakest at 3.0 g/kg. The relationship between LBE’s radiation protective effect and dosage requires further clarification. Additionally, the concentration of SCFAs was not measured in the experiments, and the direct association between metabolic pathways and radiotherapy efficacy lacks clinical data support.

Urolithin A is a byproduct of ellagitannins, which are compounds present in various fruits and nuts such as pomegranates, strawberries, and walnuts. It exhibits numerous pharmacological properties, including the ability to influence estrogen and androgen receptors, as well as offering antioxidant, anti-inflammatory, and anti-aging benefits. Additionally, Uro A helps repair colon damage caused by a high-fat diet and can alter gut microbiota (Zhao et al., 2019). Studies have shown that Uro A alleviates the loss of Lgr5^+^ and the deficiency of Axis inhibition protein 2 (Axin2), thereby reducing the impact of radiation on cell proliferation and differentiation (Li et al., 2020). Compared with the control group, Uro A treatment significantly promoted crypt regeneration. Uro A also significantly alleviated the increase in 8-hydroxy deoxyguanosine (8-OHdG), a marker of DNA damage, in the small intestines of irradiated mice, downregulated downstream molecules of the p53-mediated apoptosis pathway, and improved radiation-induced intestinal damage. In conclusion, Uro A promoted the differentiation and proliferation of ISCs, enhanced their regenerative responses, improved the morphology and structure of the irradiated mouse intestine, and significantly reduced radiation-induced apoptosis in enterocytes (Zhang et al., 2021). Furthermore, Uro A helped to reduce the disruption of gut microbiota caused by radiation and lowered the levels of Shigella, which increased due to radiation exposure. This suggests that Uro A protects against radiation damage by reshaping the gut microbiota. Additionally, Uro A may serve as a potential prebiotic to enhance the effectiveness of radiotherapy and safeguard against radiation-related harm.

### 4.3 Probiotics and probiotic combination therapy

Probiotics are a group of active microorganisms that are beneficial to the host by colonizing the body and altering the composition of the flora at a particular site of the host. Probiotics play a role in immunomodulation, anti-tumor processes, etc. Research by Ciorba et al. (2015) showed that appropriate doses of *Lactobacillus acidophilus* probiotics can help decrease gastrointestinal toxicity following radiotherapy, as evidenced by both clinical studies and preclinical models. *Lactobacillus acidophilus* has also been shown to be beneficial for radiation-induced intestinal mucosal damage in rats (Ki et al., 2014). In addition, probiotic supplements containing Bifidobacterium reduced chemotherapy-induced mucositis and radiotherapy-induced diarrhea (Badgeley et al., 2021). A dual-strain probiotic (*Lactobacillus acidophilus* LAC-361 and *Bifidobacterium longum* BB-536) reduced radiation-induced diarrhea at the end of treatment in patients with pelvic cancer (Demers et al., 2014). However, this experiment did not evaluate differences in efficacy between different radiotherapy sites. Regarding probiotic combination therapy, the study data indicated that, compared with the control group mice, solvent-treated mice promoted the proliferation and differentiation of awe posterior intestinal stem cells 3 days after radiation. The mixed probiotics significantly attenuated the reduction in lysozyme + Paneth cells (PC) and PAS-stained GC in the irradiated group, with a more pronounced effect than single probiotic therapy. 24 IF analysis showed that mixed probiotic treatment mitigated the disruption of TJ protein expression and enhanced the intestinal barrier function in irradiated mice. In comparison to the control group, the oral intake of probiotics significantly reduced the increase in malonaldehyde (MDA) levels caused by radiation and helped restore the lowered levels of glutathione (GSH), superoxide dismutase (SOD), and total antioxidant capacity (T-AOC) in the intestinal tissues of mice, thereby correcting the redox imbalance. The ELISA analysis indicated that the levels of IL-1b, IL-6, and TNF-α in intestinal tissues increased following radiation exposure, whereas the levels of the anti-inflammatory cytokine IL-10 decreased significantly. However, this reduction was notably counteracted by the addition of probiotics (Xie et al., 2024b), which reduced the inflammatory response in irradiated mice. The mixed probiotic group promoted the relative abundance of radiation protection-associated Trichosporonaceae at the family, genus, and species levels compared to the single-strain group. It also partially restored the diversity and composition of the gut microbiota and increased the abundance of SCFA-producing bacteria in irradiated mice. The probiotic mixture also encouraged the increase of anti-inflammatory and radiation-resistant bacterial metabolites sourced from gut microbiota, including SCFA, indole derivatives, and other metabolites from gut microbiota groups (Yu et al., 2023). The metabolites most abundantly found in the feces of survivors were grouped within the tryptophan metabolic pathway. This group included indole 3-carboxaldehyde and KYNA, both of which notably enhanced survival rates and lowered clinical scores in mice subjected to radiation treatment (Guo H. et al., 2020). Bacterial metabolism in mice was enriched in the tryptophan pathway and KYNA expression was increased after treatment with a mixture of probiotics. The results indicate that combined probiotic treatments have more significant therapeutic effects at the histological level than single probiotic strains. They help reduce radiation damage by influencing gut microbiota and metabolites, alleviating inflammation, and regulating oxidative stress. This will aid in creating probiotic-based treatment approaches for clinical use.

### 4.4 Fecal microbiota transplantation

Fecal microbiota transplantation (FMT) is a therapeutic method of isolating the gut microbiota of a healthy donor and transplanting it into the patient’s gut to re-establish their gut microbiota. Radiation causes an imbalance in the microbiota, leading to a reduction in the helpful bacteria Lactobacillus and Trichoderma, but this imbalance can be corrected through FMT (Tu et al., 2024). Meanwhile, tryptophan, an essential aromatic amino acid, is considered a key metabolite in the communication between the gut microbiota and the host (Su et al., 2022). Tryptophan metabolites have been shown to have the ability to reduce pro-inflammatory cytokine responses (Wlodarska et al., 2017; Krishnan et al., 2018). Tryptophan metabolites showed significant recovery in mice receiving FMT (Tu et al., 2024). The results indicate that FMT has the potential to alleviate radiation-induced colitis by regulating downstream metabolites of the microbiota, represented by members of the tryptophan pathway. Radiation therapy causes cell damage and tissue inflammation, and Gzm B is an important component of the immune system’s cytotoxic mechanism (Wlodarska et al., 2017; Krishnan et al., 2018). The study found that FMT alleviated radiation-induced GzmB upregulation. Cyp1a1 is a cytochrome P450 (CYP1) enzyme induced by the activation of the AhR (Coelho et al., 2022). Excessive Cyp1a1 activity can deplete AhR ligands, potentially leading to increased susceptibility to pathogenic bacteria and causing IBD (Schiering et al., 2017). Compared with the control group, FMT significantly downregulated Cyp1a1 expression and alleviated the inflammatory response of radiation enteritis. This research indicates that FMT reduced the impairment of barrier function caused by radiation, which is mediated by MUC2, and helped restore the levels of tight junction proteins (Zona Occludens 1, ZO-1). This suggests that FMT can influence the abnormal inflammatory signaling pathways and barrier dysfunction in the intestinal mucosa that are triggered by radiation (Tu et al., 2024). FMT also increased the production of SCFAs, which plays an important role in mitigating radiotherapy-induced intestinal damage. Treatment with SCFA (sodium acetate, sodium butyrate, or sodium propionate), particularly sodium propionate, was found to induce long-term radioprotection, remission of gastrointestinal and hematopoietic syndromes, and a decrease in pro-inflammatory responses in mice. These effects were mediated by the release of reactive oxygen species and the attenuation of DNA damage in gastrointestinal and hematopoietic tissues (Guo H. et al., 2020). Besides SCFAs, FMT generates other metabolites like n-3 Polyunsaturated Fatty Acids (n-3PUFA). These compounds can help reverse gut microbiota disorders by boosting the diversity and quantity of beneficial bacteria, while also lowering the levels of bacteria that produce intestinal LPS and those that break down mucus. This process can lead to a reduction in inflammation and oxidative stress. Cui et al. (2017) demonstrated that FMT from a healthy donor preserved the intestinal bacterial composition of irradiated mice and improved gastrointestinal function and intestinal epithelial integrity. A preliminary study reported that FMT may be safe and effective in improving intestinal symptoms and mucosal damage in patients with chronic radiation enteritis (CRE; Ding et al., 2020).

By comparing the mechanisms of the above methods, we can see that dietary supplements mainly work by supplementing dietary fiber, vitamin D, and other nutrients, which are fermented by gut microbiota into SCFA, butyrate, and other metabolites. These metabolites regulate immune responses, enhance intestinal barrier function, and inhibit tumor cell apoptosis pathways such as PI3K and Akt to suppress cancer progression. These supplements have a wide range of sources, are highly safe, and can be obtained through daily diet or nutritional supplements.

Prebiotics primarily promote the proliferation of beneficial bacteria through selective mechanisms such as LBE and Uro A, thereby upregulating tryptophan metabolites and secondary bile acids, which have anticancer effects. They are primarily derived from natural ingredients, and their metabolic pathways are well-defined. Probiotics and combination therapy enhance intestinal repair and anti-inflammatory effects by regulating oxidative stress; their clinical application is well-established, but the colonization ability of strains is influenced by individual microbiota backgrounds, and single-strain therapies have limited efficacy, while mixed-strain formulations require optimized ratios. FMT primarily restores intestinal barrier function by reshaping the intestinal microbiota structure.

With the emergence of new methods, their limitations must also be acknowledged. For example, inulin and Uro A have provided insights into anticancer therapy, but the differences arising from the translation of animal experiments to clinical settings remain unclear. Current research is limited to mouse models, and the optimal timing and dosage safety range for human administration have yet to be determined. The specific dosage and safety of sitosterol for gastrointestinal tumors also lack large-scale clinical data support. The clinical studies with the greatest potential for translation currently focus on probiotic combination therapy and FMT. The main challenge in probiotic combination therapy lies in the competitive relationships between strains. In animal experiments, the ratio of mixed strains is an optimized result, but the human intestinal environment is complex, and competition between microbial communities may lead to an imbalance in microbial ratios. Additionally, individual differences in intestinal environments may result in unstable strain colonization. Among various strategies, FMT has become the most persuasive method for studying the relationship between bacteria and disease. However, due to the lack of standardized transplantation procedures and long-term safety data, patients receiving gut microbiota transplantation may experience treatment failure due to microbiota rejection. Additionally, most studies use humanized rodents as models, which cannot simulate the complex gastrointestinal environment of the human body. In fact, radiation exposure in animal studies is often more extreme and lethal compared to humans, and there are differences in radiation resistance and microbiota. Therefore, we need to consider other more accurate experimental methods.

People have gradually come to recognize that the gut microbiome plays a crucial role in maintaining human health, and new therapeutic strategies based on gut microbial metabolites show great promise. Dietary supplements, prebiotics, and other agents can improve gut microbiota through immune regulation and metabolite secretion, thereby enhancing the efficacy of radiotherapy and reducing adverse reactions. In addition, other novel approaches such as phages and bacteriocins can also be used to engineer the gut microbiome. These new engineered products must undergo extensive research and evaluation to explore their potential for clinical application in cancer radiotherapy.

## 5 Future perspectives

Cancer is a serious human security issue that is difficult to cure due to its complexity. Whilst current anticancer drugs have been shown to have a role in providing palliative or curative treatments, there are still a variety of adverse effects that lead to reduced efficacy and poor prognosis. In recent years, as we have gained a deeper understanding of the gut microbiota, we have found that imbalances in the gut microbiota and its metabolites have been linked to a variety of diseases, including cancer. The microbiome plays a role in cancer development and treatment via various pathways, but our knowledge of this intricate system remains limited. This is largely because the micro-ecosystem is made up of a wide variety of microorganisms that are governed by complex signaling networks. As a result, distinguishing between pro- and anti-tumorigenic strains within the intricate colonies of gut microbiota is challenging. There is a need for more research to clarify the roles of specific microbial elements in inflammation, metabolism, and tumor development, as well as their connections to distant organs. Additionally, understanding the complex interactions between host responses and the microbiota is essential. Simultaneously, microbial metabolites do not exclusively encourage or hinder tumor growth, and numerous microbial metabolites can have contrasting effects depending on the context. We also need to consider synergistic or antagonistic interactions between metabolites and not just focus on the biological function of a particular metabolite. In addition, bacteria inhabiting other organs such as the oral cavity and vagina, as well as fungi and viruses, are also closely related to the development and treatment of cancer and need to be further investigated.

Currently, various microbial-based therapies, such as probiotics, FMT, antibiotics, microbial metabolites, and natural compounds, have been used as potential strategies for the treatment of radiation-induced injuries and have accumulated some evidence in clinical trials. However, concerns remain about the safety of gut microbiota group modulation strategies because the poor efficacy, complications and potential tumor protective effects are unknown. Therefore, more preclinical studies and prospective clinical trials are needed to assess the efficacy of different strategies. In terms of experimental design, a multicenter, large-sample randomized controlled trial should be adopted, with strict control of sample size to ensure balanced baseline characteristics between groups, thereby minimizing confounding biases. High-throughput sequencing technology should be employed to dynamically monitor changes in the microbiome before, during, and after radiotherapy. Longitudinal analysis should be conducted to examine the dynamic evolution of the composition and structure of microbiome metabolites, and statistical methods should be used to investigate the relationship between the microbiome and treatment efficacy, in conjunction with clinical efficacy indicators. Additionally, specific designs can be tailored to address key issues, such as collaborative experimental designs for immunotherapy, to explore the enhanced efficacy of immunotherapy following radiotherapy-induced regulation of the microbiome. Finally, for experimental patients, long-term monitoring and follow-up should be conducted, systematically recording acute and long-term adverse reactions, and assessing treatment safety through regular collection of biological samples.

Regarding technical challenges in experimental design, specifically, during the sampling process, there may be differences in gut microbiota between different intestinal segments and those caused by radiation therapy-induced intestinal damage. Additionally, the sampling process is prone to contamination, which can interfere with the interpretation of results. It is essential to thoroughly analyze potential biases that may arise during sample collection, such as sampling time. The choice of sample type can also affect detection results. Metabolites are unstable, and if not promptly subjected to low-temperature processing or frozen storage after sampling, they may degrade due to enzymatic hydrolysis or oxidation, leading to metabolite loss. Differences in analytical results may also arise from variations between laboratories and testing platforms. We must consider these technical limitations and take measures to mitigate them to enhance experimental accuracy and clinical applicability. In experiments, the anatomical location of the sampling site should be clearly marked in the sampling protocol to ensure comparability of intestinal segment origins across different samples. To prevent contamination from external impurities, strict adherence to aseptic procedures is required, and a standardized sampling time window should be established. Additionally, by implementing standardized experimental protocols and regularly calibrating instruments, technical deviations can be minimized from the source, enhancing the reliability and stability of experimental data, thereby providing more reliable evidence for clinical translation of research findings.

In addition, individual differences observed in response to radiation and the severity of radiation-related toxic effects are a major challenge in the use of radiotherapy, and the components of an individual’s gut microbiota are highly variable. From a host genetic perspective, genetic differences among individuals can influence the colonization and composition of the gut microbiota. Genetic polymorphisms may affect an individual’s susceptibility to specific microbial communities, thereby impacting the balance of the gut microbiome and ultimately influencing the response to radiotherapy. Patients with specific genetic backgrounds may exhibit differing tolerance or sensitivity to radiotherapy, potentially leading to variations in treatment efficacy. Certain genetic characteristics may make a patient’s gut microbiota more susceptible to damage during radiotherapy, potentially triggering side effects such as radiation enteritis; conversely, other genetic factors may stabilize the gut microbiota, maintaining relative homeostasis during radiotherapy, thereby helping to mitigate adverse reactions and enhance treatment efficacy.

The role of the microbiome in cancer is environment-dependent, and we must consider factors that regulate the gut microbiota, including diet, antibiotic use, geographic location, and certain treatments. Only by fully understanding these interactions can we improve the efficacy of radiotherapy and suppress adverse reactions.

For example, a long-term high-fiber diet can promote the production of beneficial bacteria, such as bifidobacteria and lactobacilli, which produce metabolites such as SCFAs, playing a positive role in intestinal health and immune regulation. Individuals following a high-fiber diet may experience reduced adverse reactions to radiotherapy and a lower risk of radiation enteritis during treatment. Conversely, a diet high in fat, sugar, and low in fiber may lead to reduced gut microbiota diversity, increased proportions of harmful bacteria, and disrupted gut microbiota balance. Similarly, during radiotherapy, the gut microbiota may be unable to effectively respond to treatment-induced stress, potentially exacerbating side effects and impairing treatment efficacy. Environmental factors such as climate, soil, and water sources in different regions can also lead to differences in the types of microorganisms people are exposed to, affecting microbiota colonization. In tropical regions, due to warm and humid climates and abundant microbial diversity, residents’ gut microbiota may also exhibit higher diversity; whereas in cold and dry regions, gut microbiota diversity may be relatively lower.

Despite these limitations, various aspects of the gut microbiota can be used as biomarkers for cancer prediction and prognosis. The sensitivity and specificity of candidate biomarkers should be validated through multicenter, large-scale studies to determine whether they can serve as universal prognostic indicators. For patients with a fragile gut microbiota that is susceptible to radiation therapy-induced damage, pre-treatment measures such as probiotic supplementation and dietary adjustments can be implemented to optimize the gut microbiota. During radiotherapy, continuous monitoring of the dynamic changes in the gut microbiota should be conducted, and treatment strategies should be adjusted based on the real-time status of the microbiota. Radiotherapy is increasingly being combined with molecular targeted therapy or immunotherapy for the treatment of solid tumors. If testing reveals that a patient’s gut microbiota characteristics indicate poor radiotherapy efficacy, immunotherapy or targeted therapy can be combined with radiotherapy. It is essential to study how the gut microbiota participates in synergistic therapy to enhance radiation-based cancer combination therapy. With the continuous advancement of medicine, it is believed that the maximum benefits of strategies such as the gut microbiota and probiotics can be realized in the future. By integrating multi-omics approaches, such as genomics and metabolomics, the interaction network between the gut microbiota, host, and tumor can be comprehensively analyzed to identify key regulatory points. By detecting and targeting the regulation of the gut microbiota and its related signaling pathways, customized probiotic formulations and prebiotic formulas have been developed to truly achieve individualized radiotherapy while reducing the complications of radiation damage.
